# Exploring the Latent Information in Spatial Transcriptomics Data via Multi‐View Graph Convolutional Network Based on Implicit Contrastive Learning

**DOI:** 10.1002/advs.202413545

**Published:** 2025-04-30

**Authors:** Sheng Ren, Xingyu Liao, Farong Liu, Jie Li, Xin Gao, Bin Yu

**Affiliations:** ^1^ School of Data Science Qingdao University of Science and Technology Qingdao 266061 China; ^2^ School of Computer Science Northwestern Polytechnical University Xi'an 710072 China; ^3^ College of Mathematics and Physics Qingdao University of Science and Technology Qingdao 266061 China; ^4^ Computational Bioscience Research Center (CBRC) Computer, Electrical and Mathematical Sciences and Engineering Division King Abdullah University of Science and Technology (KAUST) Thuwal 23955 Saudi Arabia; ^5^ School of Artificial Intelligence and Data Science University of Science and Technology of China Hefei 230026 China

**Keywords:** spatial transcriptomics, spatial domain identification, graph neural network, multi‐view learning, implicit contrastive learning

## Abstract

Latest developments in spatial transcriptomics enable thoroughly profiling of gene expression while preserving tissue microenvironment. Connecting gene expression with spatial arrangement is key for precise spatial domain identification, enhancing the comprehension of tissue microenvironments and biological processes. However, accurately analyzing spatial domains with similar gene expression and histological features is still challenging. This study introduces STMIGCL, a novel framework that leverages a multi‐view graph convolutional network and implicit contrastive learning. First, it creates neighbor graphs from gene expression and spatial coordinates, and then combines these with gene expression through multi‐view learning to learn low‐dimensional representations. To further refine the obtained low‐dimensional representations, a graph contrastive learning method with contrastive enhancement in the latent space is employed, aiming to better capture critical information in the data and improve the accuracy and discriminative power of the embeddings. Finally, an attention mechanism is used to adaptively integrate different views, capturing the importance of spots in various views to obtain the final spot representation. Experimental data confirms that STMIGCL significantly enhances spatial domain recognition precision and outperforms all baseline methods in tasks such as trajectory inference and Spatially Variable Genes (SVGs) recognition.

## Graphical abstract

STMIGCL is an implicit contrastive learning‐based multi‐view graph convolutional network framework designed for downstream tasks such as spatial domain recognition, trajectory inference, and spatially variable gene identification. By combining multi‐view learning with contrastive learning and employing contrastive learning methods that enhance contrast in the latent space, STMIGCL leads to more accurate clustering results and smoother boundary delineation.

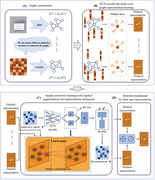

## 1 Introduction

The tissues of organisms consist of diverse cell types, and the spatial distribution of these cells within the tissue is closely related to the functions they perform. This close integration of cellular gene expression with spatial distribution offers fresh perspective for understanding tissue characteristics and disease pathology in depth.^[^ ^1^, ^2^, ^3^ ^]^ Spatial transcriptomics technology captures not just cell gene expression but also accurately locates their spatial coordinates. Since its introduction in 2016, the field of spatial transcriptomics^[^ ^4^ ^]^ has advanced rapidly, witnessing ongoing enhancements in both resolution and throughput. Current spatial transcriptomics technologies are mainly categorized into two groups, the first classification is imaging‐based techniques, including methods based on in situ hybridization and in situ sequencing, such as MERFISH,^[^ ^5^, ^6^, ^7^, ^8^ ^]^ seqFISH,^[^ ^9^ ^]^ STARmap.^[^ ^10^ ^]^ These techniques allow for the concurrent whole‐genome analysis of hundreds of genes throughout a single cell with subcellular resolution. The second classification is spatial barcoding technologies, which use non‐genome‐specific probes to capture positional barcode mRNA, storing spatial context within the barcodes, such as 10x Visium,^[^ ^11^ ^]^ Slide‐seq,^[^ ^12^ ^]^ Slide‐seqV2.^[^ ^13^ ^]^ For a more profound comprehension of tissue structure and diversity, facilitating biological insights into cell‐to‐cell communication and the microenvironment, deciphering the differences in tissue regions and cells within their original spatial context is crucial.

In spatial transcriptomics research, an important step is to identify areas exhibiting similar spatial expression patterns. A method for understanding spatial domains involves grouping sequencing spots, efficiently leveraging both gene expression data and spatial positioning to accurately assign captured spots or cells to distinct domains.^[^ ^14^, ^15^ ^]^ Currently, there are two commonly used clustering methods in spatial domain recognition: non‐spatial clustering approaches and spatial clustering approaches. The limitations of spatial transcriptomics technologies, such as low spot counts, high sparsity, and high dimensionality, constrain conventional non‐spatial clustering methods like k‐means,^[^ ^16^ ^]^ Louvain,^[^ ^17^ ^]^ and Seurat.^[^ ^18^ ^]^ These methods usually rely on gene expression information for clustering. Therefore, the domains ascertained via these techniques often exhibit discontinuity and fail to fully utilize spatial information to detect cells that might be colocalized within the same domain.

Recent advances in methodologies that integrate spatial position information with gene expression profiles have improved spatial domain recognition. For instance, utilizing a fully Bayesian statistical model, BayesSpace^[^ ^19^ ^]^ serves as a method that aims to refine imaging resolution and enhance cluster analysis. SpaGCN^[^ ^20^ ^]^ is built upon spatial data to form a neighborhood graph, followed by efficient aggregation of gene expression data from neighboring points using Graph Convolutional Networks (GCN). DeepST^[^ ^21^ ^]^ integrates gene expression with spatial locations in spatial transcriptome data through the utilization of GCNs, and concurrently extracts image features from histological data, enabling the identification of spatial domains that exhibit consistent patterns of gene expression. CCST^[^ ^22^ ^]^ combines gene expression data from individual spots with intricate global spatial information using a GCN model, enabling more accurate identification and analysis of clustering patterns in space. STAGATE^[^ ^23^ ^]^ utilizes a graph attention autoencoder to combine spatial position and gene expression data, so as to accurately recognize and analyze complex spatial domain structures. These methods frequently demonstrate less‐than‐ideal clustering results, with identified domain boundaries frequently appearing fragmented and misaligned with pathological annotations. The recently proposed Spatial‐MGCN^[^ ^24^ ^]^ utilizes a multi view graph convolutional network with the attention mechanism to effectively model gene expression and spatial location. PAST^[^ ^25^ ^]^ proposes a self‐attention framework built on priors, serving as a variational graph convolutional autoencoder tailored for ST data. SEDR^[^ ^26^ ^]^ employs a Variational Graph Auto‐Encoder (VGAE) approach to merge spatial neighborhood connections into point representations, facilitating spatial domain recognition tasks. These methods presume that neighboring points share the same type during graph construction, an assumption that is not always accurate. Furthermore, some methods utilizing contrastive learning have been proposed. By combining contrastive learning with graph neural networks, the performance of spatial domain recognition has been further enhanced. Among them, conST^[^ ^27^ ^]^ utilizes contrastive learning methods, introducing a two‐stage contrastive learning framework to acquire a low‐dimensional embedding that is representative. By randomly shuffling spatial representation graphs, SpaceFlow^[^ ^28^ ^]^ skillfully employs a contrastive learning strategy to generate negative samples, thereby enhancing the learning efficacy of deep graph neural networks. ConGI^[^ ^29^ ^]^ also adopts contrastive learning strategies to adapt gene expression to histopathological images, achieving spatial clustering. GraphST^[^ ^30^ ^]^ method serves as a novel spatial clustering framework that utilizes a contrastive learning strategy. By integrating the methods of GCNs and self‐supervised contrastive learning, GraphST aim to minimize the distance between embedding vectors to enhance the performance and accuracy of the model. However, existing methods based on contrastive learning rely on augmentation techniques driven by random perturbations, which may alter the original semantic information and result in poor clustering performance.

To address the aforementioned issues, we present STMIGCL, a multi‐view graph convolutional network framework built upon implicit contrastive learning. Specifically, we initially create multiple neighbor graphs utilizing gene expression profiles and spatial location data, and each neighbor graph represents a specific graph structure. For each neighbor graph, we train a VGAE aimed at reconstructing the graph topology. We leverage the latent distribution obtained from VGAE for contrastive learning, treating the vector representation of a node and its latent augmentation as positive pairs, while considering other pairs as negative. This augmentation method significantly enhances the effectiveness of contrastive learning, enabling it to adaptively preserve the core semantic information of graph data without the need for arbitrary manual designs or reliance on prior human knowledge. Compared to contrastive methods that only rely on simple random augmentations, this approach demonstrates superior data insight, effectively capturing and leveraging the key information within the dataset, thus significantly improving the discriminative ability of the learned representations. Concurrently, we employ GCN to obtain latent representations for each view. By integrating multi‐view learning with contrastive learning, we not only completely harness the unique strengths of individual GCN to deeply explore the latent information within each view, but also effectively integrate and leverage the complementary advantages across different views, resulting in more comprehensive and enriched information acquisition. By further leveraging the aforementioned contrastive approach, we can significantly enhance the discriminative power of the representations, ultimately optimizing clustering performance. Finally, to achieve a more efficient fusion of these views, we apply an attention mechanism that dynamically integrates the representations from each perspective, leading to the final spot representation. Throughout this process, we train the model utilizing both reconstruction loss and implicit contrastive loss. Then, we leverage the obtained spot representations for downstream applications encompassing spatial domain recognition, trajectory inference, and identification of Spatially Variable Genes (SVGs).

## 2 Results

### 2.1 Overview of STMIGCL

**Figure 1:**
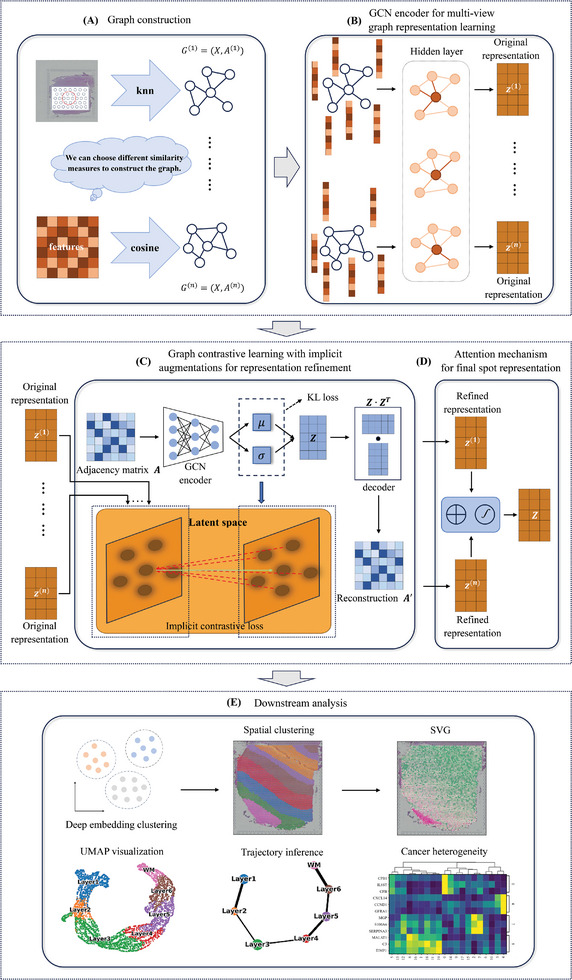
Overview of STMIGCL method. A) Based on spatial transcriptomics data, STMIGCL constructs multiple adjacency graphs using different similarity metrics. B) For each view, STMIGCL integrates gene expression and neighbor graphs using GCN to capture latent representations of different views. C) To further refine the learned representations, STMIGCL trains VGAE aimed at reconstructing the graph's topological structure. By utilizing improvements from the latent space learned by VGAE as a contrastive resource, it further refines the representations from each view through training with an implicit contrastive loss. D) STMIGCL incorporates an attention mechanism to dynamically combine the representations from various views, thus deriving ultimate spot representations that capture the importance of different views. E) The resulting spot representations from STMIGCL are applicable to a range of downstream analysis tasks, such as clustering, trajectory inference, SVGs identification, etc. Among them, an unsupervised iterative clustering approach is employed in the spatial domain recognition task to group distinct spots into separate clusters.

STMIGCL is a multi‐view graph convolutional network framework based on implicit contrastive learning method, which can effectively extract the latent information in spatial transcriptomics data. **Figure** **1** provides a complete illustration of the entire STMIGCL workflow. As shown in Figure 1A, by utilizing the gene expression profiles and the spatial location information from spatial transcriptomics data, we construct multiple neighbor graphs using different methods, aiming to extract richer and more effective information from the data through collaborative learning across different views. Then, we use GCN to learn the low‐dimensional representations of each view, where *Z* ^(*i*)^ signifies low‐dimensional embedding of *i*‐th view, aggregating both graph structure and gene expression features (Figure 1B). As mentioned earlier, we further incorporate contrast learning mechanism to enhance the informativity and discriminability of the obtained representations (Figure 1C). Distinct from prior research, we utilize the latent space derived from VGAE as the foundation for contrastive enhancement. Through the training of a contrastive loss, our model's learned representations are designed to not only preserve the graph's inherent semantic content, but also exhibit enhanced discriminative. To capture the significance of various view embeddings, we incorporate an attention mechanism that facilitates their adaptive integration. Therefore, we not only deeply mine the potential information in the original data from different perspectives through multi‐view, but also use contrast learning to make the obtained representation more discriminative. At the same time, the attention mechanism effectively fuses the representations of different views, so that the final spot representation can not only effectively retain the semantic information in the original data, but also be more informative and discriminative (Figure 1D). Finally, the learned spot representations will be utilized in subsequent tasks encompassing spatial domain recognition, trajectory inference, and SVGs recognition (Figure 1E).

### 2.2 STMIGCL Performs Spatial Clustering on the 10x Visium Dataset of Human Dorsolateral Prefrontal Cortex, Improving the Recognition of Known Layers

**Figure 2:**
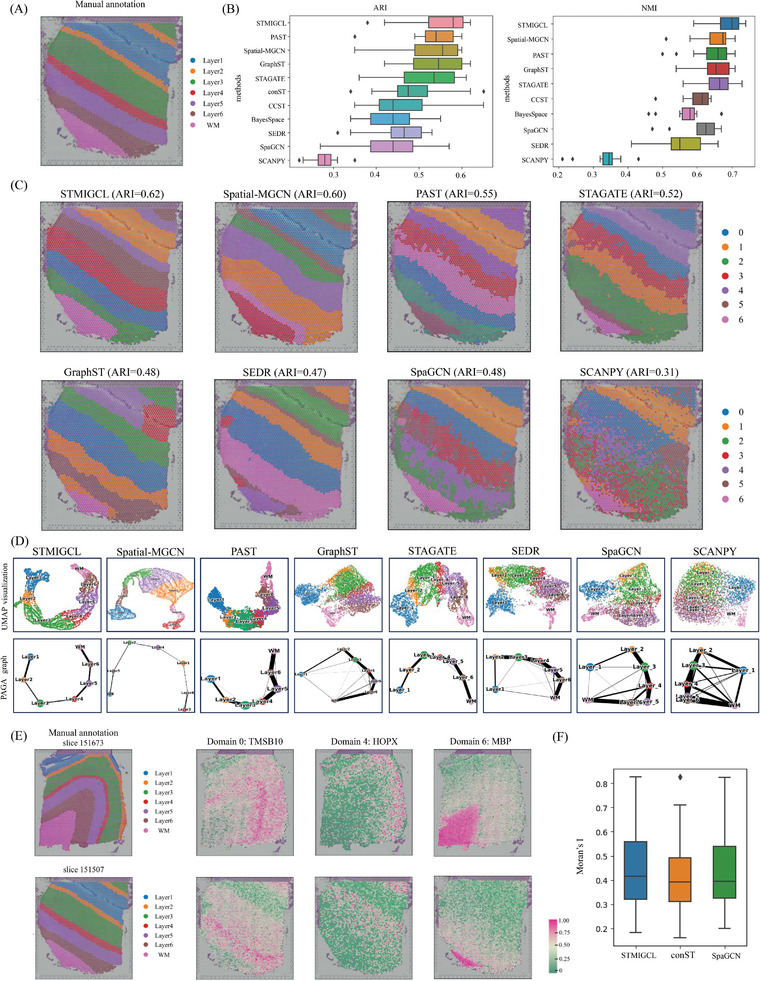
STMIGCL performs spatial clustering on the human dorsolateral prefrontal cortex (DLPFC), improving the identification of known layers. A) The manually annotated ground truth for the cortical layers and white matter of DLPFC section #151 507. B) Using ten different methods to cluster all 12 slices of the DLPFC dataset, the boxplot is constructed based on ARI and NMI scores to illustrate the accuracy of clustering. C) Cluster assignments produced by SCANPY, SpaGCN, SEDR, GraphST, STAGATE, PAST, Spatial‐MGCN, and STMIGCL in section #151 507 of the DLPFC. D) Section #151 507 of the DLPFC is utilized to generate UMAP visualizations and PAGA graphs, which are derived from embeddings produced by SCANPY, SpaGCN, SEDR, STAGATE, GraphST, PAST, Spatial‐MGCN, and STMIGCL. E) The spatial expression patterns observed for SVGs in slices #151 673 and #151 507 by STMIGCL suggest the potential transferability of these SVGs to various brain regions. F) Boxplot was created to illustrate the Moran's I values for the detected SVGs, utilizing three distinct methods.

In this research, we initially evaluates the spatial clustering capabilities of STMIGCL by utilizing the 10x Visium dataset of human dorsolateral prefrontal cortex (DLPFC) slices.^[^ ^31^ ^]^ Guided by morphological characteristics and gene markers, Maynard et al. manually labeled the layers of DLPFC and WM, as shown in **Figure** **2**A. Based on this, this study tested the ability of STMIGCL and baseline methods PAST,^[^ ^25^ ^]^ Spatial‐MGCN,^[^ ^24^ ^]^ GraphST,^[^ ^30^ ^]^ STAGATE,^[^ ^23^ ^]^ CCST,^[^ ^22^ ^]^ conST,^[^ ^27^ ^]^ BayesSpace,^[^ ^19^ ^]^ SEDR,^[^ ^26^ ^]^ SpaGCN,^[^ ^20^ ^]^ and SCANPY^[^ ^32^ ^]^ performed in an unsupervised manner to reconstruct annotated cortical anatomy. The Supplementary Notes 1 include descriptions of the relevant baseline methods.

In this article, two evaluation indicators have been selected to comprehensively and accurately assess the clustering performance of various methods. For detailed information, please consult the methods section. In all 12 slices, STMIGCL achieves the best clustering performance, obtaining the highest median scores for both ARI and NMI values, as illustrated in Figure 2B. Specifically, the median ARI score of STMIGCL is 0.58, which is 0.04 and 0.03 higher than those of the sub‐optimal methods, PAST and Spatial‐MGCN, respectively. Similarly, by analyzing the NMI results of all methods, it can also be found that the proposed method obtains the highest NMI value, which is 0.03 higher than the suboptimal method. The aforementioned results have proven that the method proposed in this paper, by ingeniously integrating multi‐view learning with contrastive learning techniques, significantly enhances the informativeness and discriminative capability of the obtained latent representations. This innovation not only further improves the accuracy of clustering results but also makes the delineation of clustering boundaries more delicate and smoother, demonstrating the remarkable effectiveness and unique advantages of this method in enhancing clustering performance. Furthermore, by contrasting the performance of ten clustering methods that integrate spatial information with one clustering method that does not consider spatial information, we can clearly see the significant advantages of the former in the recognition process. Fully considering spatial information in the process of spatial domain recognition is further emphasized by this observation. We also visualized the differences in ARI and NMI values obtained through all methods to further validate the performance of the proposed model in this study, that can be found in the Figure S3 (Supporting Information).

Next, we use a slice (#151 507) from this dataset to further illustrate the efficacy of each approach in spatial domain identification tasks. The clustering visualization results are shown in Figure 2C. In this slice, the non‐spatial clustering method SCANPY performs the worst, only able to recover clustering of the first layer. SpaGCN can better separate the first and second layer structures, but fails to correctly recover layers 3–6 as well as the WM. SEDR, STAGATE and GraphST methods identify domains closer to the manually labeled tissue layers, but the obtained layer thickness is inaccurate. The PAST method faces challenges in accurately identifying the third layer and exhibits noticeable inconsistencies when delineating domain boundaries, particularly between the third and fifth layers. In comparison, the Spatial‐MGCN model performs similarly to the approach presented in this paper, successfully identifying most of the discernible domains. However, it still shows deviations in accurately defining the fourth and fifth layers, with boundaries not aligning perfectly with manual annotations. The STMIGCL model stands out for its high clustering accuracy, producing clear and exceptionally smooth domain boundaries. This result underscores an important trend: methods that incorporate multi‐view learning (such as STMIGCL and Spatial‐MGCN) or contrastive learning (such as STMIGCL and GraphST) tend to produce smoother domain boundaries in domain identification tasks, significantly reducing the occurrence of discrete points. This suggests that multi‐view learning and contrastive learning methods are particularly effective at uncovering latent information within the data, enabling them to capture more comprehensive and nuanced features, which in turn enhances the accuracy and stability of domain identification. In addition, spatial clustering methods, including STMIGCL, still perform poorly in distinguishing between the third and fourth layers. We think this may be due to a high similarity in gene expression between these two layers, making it difficult for the clustering algorithm to accurately differentiate them. The quantitative evaluation using ARI reveals that STMIGCL achieved the highest ARI value, and the clustering results for all other slices can be found in Figure S1 (Supporting Information). We further conducted a comparative analysis of the clustering performance of the model under different hyperparameter configurations (see Figure S5, Supporting Information for details). The results reveal that the model demonstrates strong robustness to variations in various hyperparameters.

Through the integration of spatial information, STMIGCL adeptly captures the distance relationships between spatial domains and portrays spatial trajectories within UMAP plots. Consequently, the low‐dimensional embeddings derived from STMIGCL are beneficial not only for clustering analysis but also offer substantial value in subsequent analytical endeavors. Taking slice #151 507 of the DLPFC as an example, as shown in Figure 2D, in the UMAP visualization embedded by the non‐spatial method SCANPY approach, spots from different layers are mixed together, and clear separation results are not obtained. Although SpaGCN, SEDR, and GraphST are able to roughly distinguish spots from different layers, the boundary mixing between layers is quite high. STAGATE and PAST also exhibit varying degrees of boundary mixing. In contrast, when generating UMAP plots based on STMIGCL and Spatial‐MGCN embeddings, we can clearly observe more consistent and coherent spatial trajectories. This trajectory not only smoothly transitions from the first layer to the sixth layer but also demonstrates good continuity in the areas involving white matter. Not only does this finding correspond with the functional resemblance among neighboring cortical layers, but more significantly, it also maintains consistency with the temporal sequence of these regions in the brain. The trajectory inference visualization results reveal that the PAGA plot, embedded by STMIGCL, demonstrate a higher resemblance between adjacent layers, allowing for trajectory inference in a sequential manner from Layer_1 to Layer_6, while results from other methods are mixed. The clearer trajectory inference results obtained through the proposed method may provide valuable insights for scientists in uncovering the deeper operational mechanisms of important brain regions, and could potentially serve as a key tool to help them gain a deeper understanding of the “logical” mysteries behind these complex brain areas.^[^ ^33^ ^]^ Figure S2 (Supporting Information) includes the UMAP visualizations and PAGA graphs for the remaining slices.

To further verify the identified spatial domains, this study continues to adopt the method of SpaGCN for detecting SVGs. In Note 2 (Supporting Information), detailed steps for detecting SVGs are described. We then compare these with the SVGs previously identified by conST and SpaGCN. Applying the methodology introduced in this study, a total of 79 SVGs were detected in slice #151 673, distributed across various domains. Among them, 40 SVGs were specific to domain 1, corresponding to white matter, while the patterns of SVGs in other domains were less clear. This result indicates that the gene expression profiles of white matter spots are significantly different from those of the neuronal layer spots, with much smaller differences observed among the six neuronal layers. We visualized three SVGs detected by STMIGCL, which marked two neuronal layers and one white matter layer, as shown in Figure 2E. Among these results, TMSB10 is widely recognized for its role in promoting cell migration.^[^ ^34^ ^]^ Through our visualization analysis, we clearly demonstrated the expression of this gene across different tissue sections, further validating the generalizability of its function. Additionally, HOPX, a unique homology domain protein, plays a critical role during development,^[^ ^35^ ^]^ and our results reaffirmed its importance. Overall, the SVGs we detected not only successfully transferred across different sections, but the domain labels they represent also showed high consistency between sections. To further validate the specificity of our method in revealing the spatial expression patterns of SVGs, we used Moran's I index to quantify the spatial autocorrelation of gene expression, as shown in Figure 2F. This quantitative analysis further confirmed the effectiveness of our approach.

### 2.3 STMIGCL Analyze the Spatial Domain of Human Breast Cancer Tissue from a Finer Level

**Figure 3:**
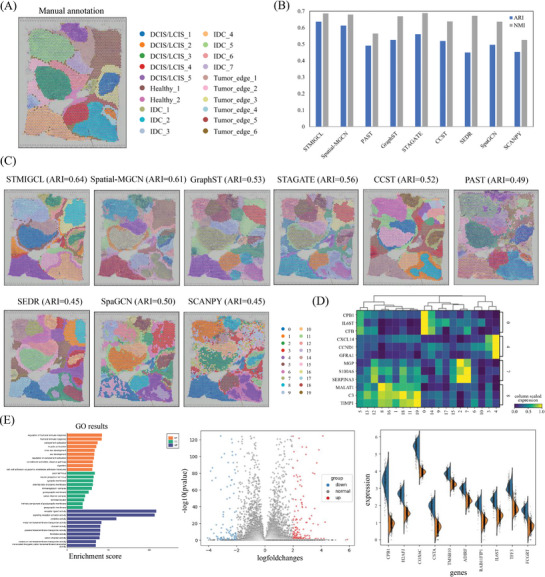
STMIGCL analyze the spatial domain of human breast cancer tissue from a finer level. A) The manually annotated human breast cancer dataset, utilizing H&E staining for its classification. B) Bar graph depicting ARI and NMI for STMIGCL alongside eight comparison methods. C) The human breast cancer data underwent clustering analysis utilizing various methods, including Spatial‐MGCN, GraphST, STAGATE, CCST, PAST, SEDR, SpaGCN, SCANPY, and STMIGCL, each generating distinct clustering results. D) A heatmap depicting the expression of structural domains for the three most differentially expressed genes (DEGs) in clusters 0, 4, 7, and 8 is presented. E) The enrichment analysis and differential expression analysis between cluster 0 and cluster 4 are presented. The bar chart displays the GO results between cluster 0 and cluster 4, and the volcano plot illustrates the DEGs between these clusters, while the violin plot displays the differential expression of the top 10 genes between cluster 0 and cluster 4.

In this study, a dataset of human breast cancer tissue,^[^ ^36^ ^]^ obtained from the 10x Visium platform, is subject to further analysis. **Figure** **3**A shows the manual annotation results of Xu et al.^[^ ^26^ ^]^ Among all methods, STMIGCL achieves the highest ARI and NMI scores, as illustrated in Figure 3B. By visualizing the clustering results obtained from all methods, it can be observed that STMIGCL, Spatial‐MGCN, GraphST, STAGATE, CCST, and SEDR can identify more distinguishable domains and obtain smoother clustering segmentation results, as shown in Figure 3C. Further analysis of the visualization results shows that STMIGCL can distinguish the IDC domain and most of the DCIS/LCIS domains. Although Spatial‐MGCN has an ARI value similar to STMIGCL, it struggles with delineating the IDC domains and their boundary regions. In contrast, STAGATE exhibits significant clustering confusion and a large number of outliers. Except for the IDC_4 domain and parts of the DCIS/LCIS domains, it fails to effectively separate most of the distinguishable domains. While GraphST's ARI score is similar to that of CCST, it cannot accurately distinguish most of the identifiable domains. The clustering results obtained by CCST are even more chaotic, failing to sufficiently separate healthy tissue and unable to classify them into distinct clusters. This discrepancy could stem from GraphST, STAGATE, and CCST primarily emphasizing local spatial arrangement while neglecting the interrelationships between different tissue regions. As for PAST and SpaGCN, although their results are similar, the clustering results are clearly more disordered, with spots from different domains intertwining, making them difficult to distinguish. SEDR and SCANPY yield the same results, but SEDR performs better in terms of modularity, although the classification of most of its points is not accurate.

To analyze the heterogeneity of cancer tissues, this study contrasted the expression levels of the leading three differentially expressed genes (DEGs) from clusters 0 (DCIS/LCIS), 4 (IDC), 7 (Tumor edge), and 8 (Health) across all structural domains, revealing notable heterogeneity among these clusters, as shown in Figure 3D. Furthermore, we performed enrichment analysis and differential expression analysis comparing Cluster 0 (DCIS/LCIS) and Cluster 4 (IDC) to delve deeper into the gene expression disparities between DCIS/LCIS and IDC. Figure 3E presents the findings of this analysis. At last, we identified 249 significant DEGs (|logFoldChange|≥2&&‐log10(P‐value)<0.05) between these two clusters. Among the detected notable DEGs, we found that the detected CPB1 is closely related to DCIS.^[^ ^37^ ^]^ DCIS is considered a precursor state preceding the development of IDC.^[^ ^38^, ^39^ ^]^ However, accurately distinguishing atypical ductal hyperplasia (ADH) from low‐grade DCIS remains challenging, leading to numerous women under‐going surgical procedures that may not be necessary. Available data suggests that untreated DCIS has a potential to progress into IDC in a range of 14% to 60% of cases, which highlights the significance of discerning the genetic characteristics of DCIS. In cluster 0 (DCIS/LCIS), high expression of CPB1 was proved to be associated with better survival results. It can predict the majority of DCIS cases, helping to distinguish between DCIS and ADH or IDC, and predict whether DCIS may progress to IDC. Upregulation of CPB1 also suggests that cluster 0 has a higher metastatic potential. In addition, the detected COX6C has also been shown to have close associations with various diseases and plays a pivotal role in the emergence of resistance to anticancer drugs in neoplastic cells.^[^ ^40^ ^]^ The up‐regulation of COX6C may indicate the enhancement of drug resistance in cancer cells. On the other hand, cluster 4 (IDC) exhibits the suppressive influence of the chemokine CXCL14 on tumor growth and metastasis in human breast cancer cells, as evidenced in both in vitro and in vivo studies. Additionally, the expression levels of this factor are significantly associated with both the survival rates of breast cancer patients and the occurrence of lymph node (LN) metastasis. CXCL14 has been demonstrated to be a suppressor of breast cancer growth and metastasis.^[^ ^41^ ^]^ Upregulation of CXCL14 may provide new strategies for future breast cancer treatment. We further verified the differential gene expression patterns in cluster 0 (DCIS/LCIS region) and cluster 5 (DCIS/LCIS edge), that can be found in the Figure S4 (Supporting Information). In conclusion, STMIGCL enables a more precise dissection of cancer tissue heterogeneity, which helps researchers to enhance their understanding of ST data and provide a theoretical basis for formulating targeted treatment strategies.

### 2.4 STMIGCL Spatial Clustering more Accurately Delineated the Tissue Structure of Stereo‐seq Data

**Figure 4:**
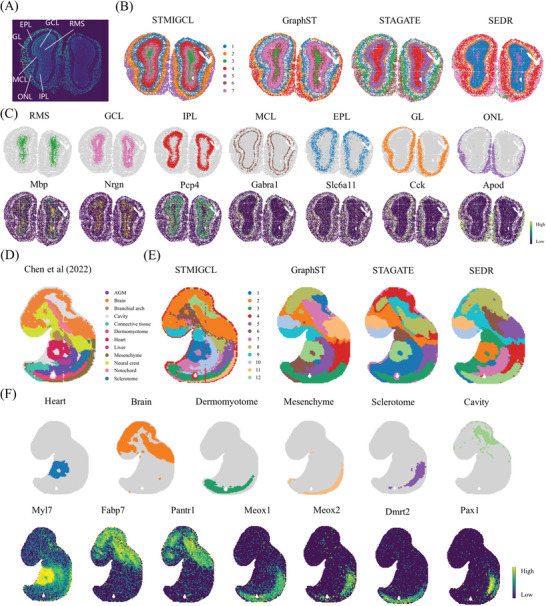
STMIGCL spatial clustering more accurately delineated the tissue structure of Stereo‐seq data. A) The laminar organization of the mouse olfactory bulb. B) The spatial domain visualization results recognized by SCANPY, STAGATE, GraphST, and STMIGCL. C) The visualization results of each structural domain identified by STMIGCL, as well as the visualization results of the corresponding marker gene expression. D) The annotations of tissue structural domains were derived from the original Stereo‐seq investigation of E9.5 mouse embryos. E) The outcomes of clustering by SEDR, STAGATE, GraphST, and STMIGCL on the dataset pertaining to E9.5 mouse embryos are presented. F) The visualization of specific spatial domains recognized by STMIGCL, along with the expressions of their respective marker genes, is exhibited.

Besides the 10x Visium platform, this study extends the validation of STMIGCL's spatial domain recognition capabilities to the coronal mouse olfactory bulb tissue dataset acquired from Stereo‐seq.^[^ ^42^ ^]^ The manual annotation results are shown in **Figure** **4**A.

By comparing the clustering results of STMIGCL with the baseline methods GraphST, STAGATE and SEDR, it can be observed that STMIGCL can accurately discern the laminar tissue of this dataset and obtain results that match the annotated distribution, as shown in Figure 4B. Further analysis shows that although STAGATE effectively separate the three structures (ONL, GL, EPL) of the outer layer, the clustering results obtained by this method exhibit high inter‐cluster mixing and unclear boundaries, while SEDR mixes the three structures of the outer layer together. For the inner layer, neither STAGATE nor SEDR clearly identified the three inner structures (IPL, GCL, RMS). Although GraphST successfully discriminates among the three structures in the inner layer, it exhibits inter‐cluster mixing when partitioning the outer layer's three structures. Next, we employed marker genes specific to each anatomical region to verify the findings of STMIGCL. Upon validation, it becomes evident that the identified clusters demonstrate a notable correspondence with the expression patterns of previously reported marker genes, including Mbp, Pcp4, and Nrgn,^[^ ^43^, ^44^, ^45^ ^]^ as shown in Figure 4C. For certain marker genes, like Mbp and Pcp4, exhibit overlapping high expression levels in adjacent areas, which is anticipated given the commonality of cell types across diverse structural regions within organs. Since similar cell types tend to share markers, this overlap in expression patterns is consistent with known biological principles. In summary, STMIGCL can utilize both whole‐transcriptome information and spatial positions to identify pertinent anatomical regions.

In addition, this research also used the Stereo‐seq dataset of E9.5 mouse embryos to verify the model performance, which measured 23015 genes in 5913 cells. The tissue domain annotations were derived from the primary study, with a total of 12 regions labeled, including AGM, branchial arches, brain, cavity, dermomyotome, connective tissue, liver, heart, mesenchyme, notochord, neural crest, and sclerotome, as shown in Figure 4D.

By visualizing the clustering results obtained by STMIGCL and the baseline methods, it can be observed that the clustering results obtained by STMIGCL can capture most of the intricate structures within the embryo and match the original annotation distribution (Its quantitative evaluation results can be found in the Table S2, Supporting Information). It effectively discerns significant regions such as the heart, brain, dermomyotome and sclerotome, as shown in Figure 4E. A crucial aspect to note is that these marker genes exhibit a remarkable concordance and coherence with the genetic sequences of established principal organs. Particularly, the heart region marked by Myl7, brain region by Fabp7 and Pantr1, dermomyotome by Meox1 and Dmrt2, mesenchyme by Meox1, Mylpf and sclerotome by Pax1 were all well matched by the clusters of STMIGCL, as shown in Figure 4F. On the contrary, the clustering results obtained by GraphST, STAGATE, and SEDR mix most of the tissue regions together. However, these methods can accurately identify the heart region. In addition, GraphST and SEDR separates part of the liver, which is not achieved by other methods.

### 2.5 STMIGCL can Accurately Partition the Laminar Structure of the Mouse Visual Cortex

**Figure 5:**
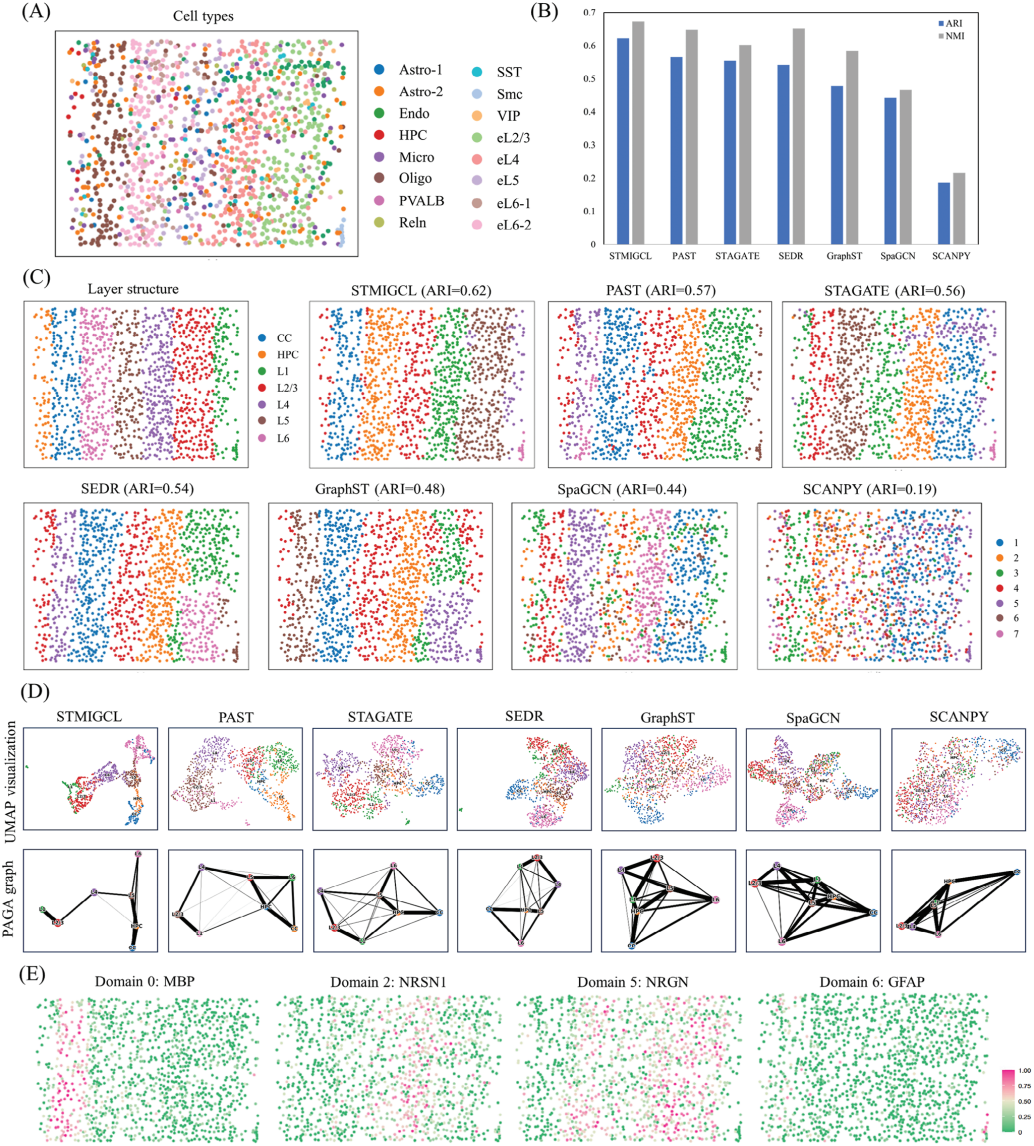
STMIGCL can accurately partition the laminar structure of the mouse visual cortex. A) The original study provided the distribution pattern of cells within tissue sections. B) Bar representations of the clustering metrics, regard the ARI and NMI, for STMIGCL and its benchmark methods. C) The originally annotated segmented layers within the tissue structure, along with the domains detected by STMIGCL, PAST, STAGATE, SEDR, GraphST, SpaGCN, and SCANPY. D) Visual representations of UMAP visualization and PAGA trajectory plots. E) The spatial expression patterns identified by STMIGCL for SVGs.

The study also conducted an analysis of a STARmap dataset, which was acquired from the mouse visual cortex and possesses single‐cell resolution. **Figure** **5**A illustrate the distribution pattern of cells within the tissue structure, while Figure 5C provides annotated segmentation layers of this tissue structure, as outlined in the original investigation.^[^ ^10^ ^]^ By comparing the clustering accuracy obtained by STMIGCL and baseline methods (as shown in Figure 5B), it can be concluded that the model proposed in this study achieved the highest ARI and NMI values, surpassing all baseline methods. By visualizing the clustering results obtained by STMIGCL and baseline methods (as shown in Figure 5C), STMIGCL is able to detect spatial domains that are more closely aligned with the annotated tissue structure. In contrast, the non‐spatial clustering method SCANPY exhibited the highest degree of mixing in laminar structures, while clustering methods that utilized spatial information performed notably superior performance compared to non‐spatial approaches. Among them, the results obtained by PAST, STAGATE and SEDR are more consistent with the annotated structures. However, further analysis revealed that PAST had numerous mixed points when delineating the L4‐L6 domains, resulting in inaccurate clustering boundaries. STAGATE obtained inaccurate layer thicknesses and had numerous outliers, which affected its clustering accuracy. SEDR did not divide the L1 and L2/L3 domains. The clustering results obtained by GraphST and SpaGCN were poorer, with ARI value of 0.48 and 0.44. The findings underscore the preeminent capabilities and distinct advantages exhibited by STMIGCL in spatial domain recognition tasks.

Next, to further illustrate the dataset's embeddings, this study employs UMAP for visualization and subsequently presents the spatial trajectories within the UMAP graph, as exhibited in Figure 5D. The experimental results show that in the UMAP graph of the STMIGCL embeddings, cell tissues belonging to different structural domains are well separated, while in UMAP graphs obtained through other methods, there is a higher degree of mixing between different domains. To further validate the results, PAGA graphs were constructed using embeddings acquired from all methods. We can observe clear developmental trajectories in the PAGA graph embedded by STMIGCL, while PAGA graphs generated by other baseline methods show different degrees of mixed developmental trajectories.

Furthermore, this study further validates the identified spatial domains by detecting SVGs. A total of 9 SVGs were detected using STMIGCL, including genes NRGN, NRSN1, MBP, and GFAP, with the visualization results shown in Figure 5E. These genes exhibited significantly enhanced expression patterns within the identified domains, highlighting their functional roles in specific spatial regions. Among them, NRGN showed particularly high expression levels in brain regions associated with cognitive functions, especially within hippocampal CA1 pyramidal neurons.^[^ ^46^ ^]^ Research has demonstrated that NRGN knockout mice exhibit notable deficits in spatial learning and display anxiety‐like behaviors. These findings underscore NRGN's pivotal role in mediating interactions between hippocampal activity, stress, and behavioral performance.^[^ ^47^ ^]^ In addition, the detected NRSN1 gene is likely closely associated with neural development. Meanwhile, the GFAP gene, as a cell‐specific marker, is believed to play a significant role in cellular communication. Notably, GFAP knockout mice exhibit marked functional impairments in reflex‐based tasks such as blink responses, further underscoring the critical importance of this gene.^[^ ^48^ ^]^ In summary, STMIGCL successfully detected the majority of SVGs in the STARmap dataset and demonstrated superior performance in distinguishing spatial domains compared to baseline methods. These findings confirm STMIGCL's robust capabilities in spatial transcriptomics research and its potential for elucidating spatial gene expression patterns.

### 2.6 Interpretability and Ablation Study of STMIGCL

The proposed model in this study, STMIGCL, mainly integrates gene expression profiles with spatial localization data to mine potential information in spatial transcriptome data. Specifically, to efficiently harness both gene expression data and spatial positioning information within spatial transcriptome datasets effectively, this study constructs different neighbor graphs by using different similarity metrics. Based on this, by integrating multi‐view learning and contrastive learning techniques, we deeply mined the potential information in the data from various perspectives. Therefore, utilizing the outcomes of deep embedding clustering, this study visualizes the graph structure of different views in multi‐view learning. By reflecting the clustering results on the graph nodes of each view, we aim to explain the model's effectiveness to a certain extent.

**Figure 6:**
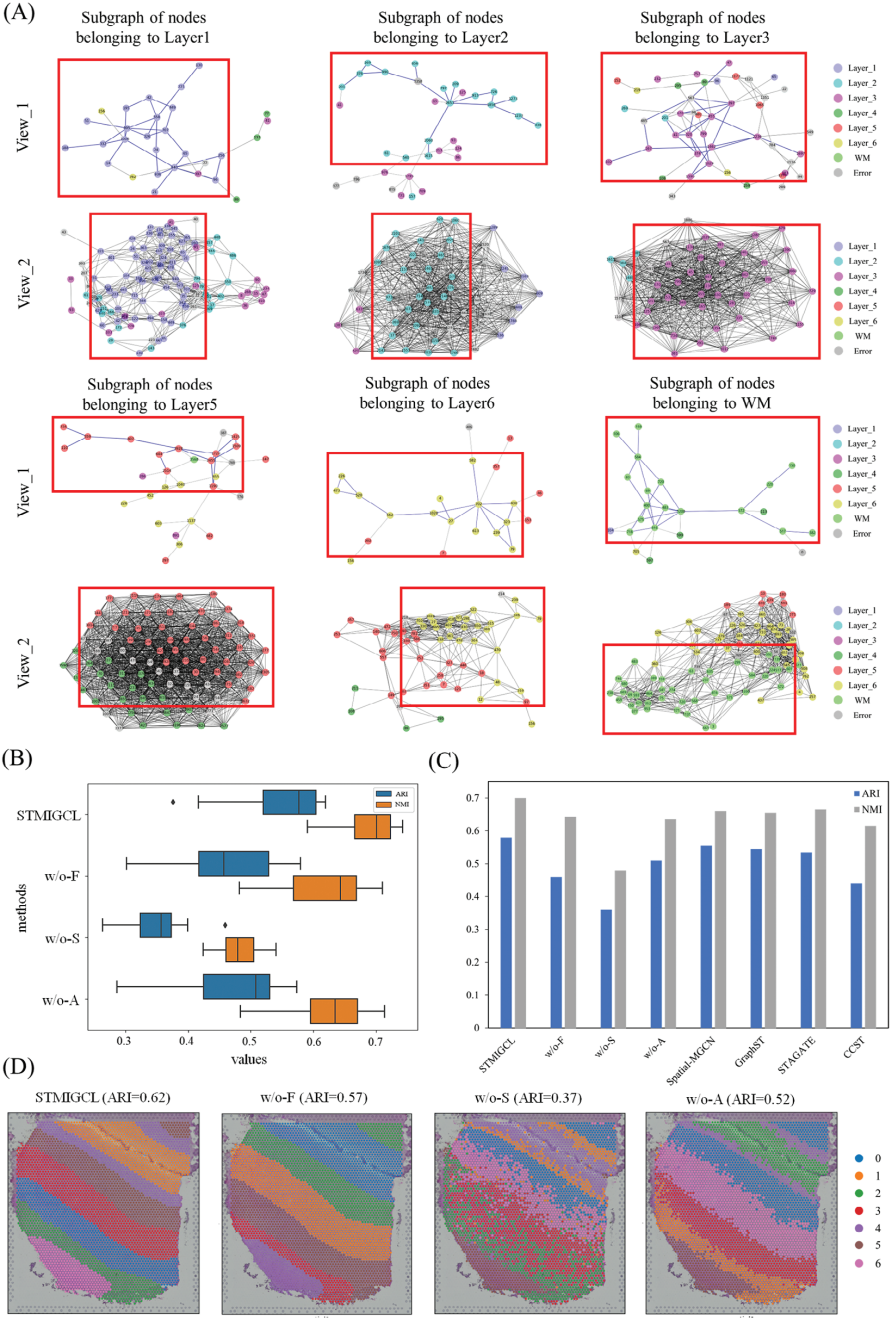
Interpretability and ablation study of STMIGCL. A) The graph structures are visualized from different perspectives based on the clustering results of various cortical spots in the DLPFC dataset (slice #151 507). View_1 represents the subgraph structure extracted from the feature view, while View_2 represents the subgraph structure extracted from the spatial view. The clustering results within the subgraphs of nodes are visualized by randomly selecting nodes belonging to different layers. The content within the red boxes represents the randomly selected nodes from the corresponding layers. B) Boxplot of ARI and NMI values for STMIGCL and its variants on the DLPFC dataset. C) The bar chart displays the average ARI and NMI values for each method. D) The visualization results of STMIGCL and its variants on slice #151 507 of the DLPFC dataset.

Selecting a slice (#151 507) from the DLPFC dataset, based on the spatial clustering results, we selected nodes belonging to different layers. The clustering results were visualized in the graph structures of different views, as shown in **Figure** **6**A. We observed that nodes belonging to different layers exhibit the same clustering results in the graph structures of different views, meaning that nodes belonging to the same cluster are able to aggregate together in different views. Further analysis of the results reveals that even if a node in the graph is incorrectly classified into different clusters, it does not affect the judgment of the model on its adjacent nodes, which further demonstrates the robustness of the model. In addition, the nodes aggregated by different neighbor graphs constructed using different similarity measures are different. This indicates that multi‐view learning can effectively mine potential information in spatial transcriptome data and aggregate node information from different perspectives.

In addition, to gain a deeper understanding of the individual components' contributions within the STMIGCL approach, this research conducts ablation experiments on the DLPFC dataset and designs three variants of STMIGCL. We first excluded the feature view and trained the model with only the spatial view as input, labeled as w/o‐F. The purpose of this experiment is to evaluate the impact of feature similarity on the effectiveness of the model, thereby revealing the contribution of the feature view to the final results. Then, we removed the spatial view to evaluate the importance of spatial coordinates in the study of spatial transcriptomics data, labeled as w/o‐S. Through this experiment, the impact of spatial information on the model's performance can be understood more intuitively. Finally, we validated the role of the attention mechanism in view fusion, labeled as w/o‐A. Through the comparative analysis of the above ablation experiments, the functions of each component in STMIGCL and their impact on the overall performance of the model can be comprehensively evaluated. The experimental findings consistently demonstrate that STMIGCL out performs its variants across two evaluation metrics, as shown in Figure 6B. Please refer to the Table S3 (Supporting Information) for detailed experimental results.

First, among the two variants that use a single view as input, w/o‐F demonstrates better performance on both metrics, achieving the highest median among all variants. However, its results exhibit a large variance, indicating a certain degree of instability in the model's performance. This result indicates that cosine similarity can effectively measure the similarity between feature vectors. By constructing a feature view, the model can better capture the underlying information in the data during the training process. However, when the feature view is removed, the model struggles to maintain stable performance due to the absence of this structural information. This finding further emphasizes the critical role of the feature view in ensuring the model's effectiveness and stability. Second, w/o‐S obtains the lowest results in both evaluation metrics, further illustrating the importance of adding spatial location information into the spatial domain recognition task. Spatial location information significantly enhances the model's ability to capture spatial structural features by providing a positional reference for the spatial distribution of the tissue. The spatial view constructed using spatial location data enables a more intuitive and comprehensive depiction of the distribution of spots across different domains, thereby revealing the spatial structure of the tissue. Variants lacking the spatial view struggle to effectively represent the relationships and distribution characteristics of spots in the spatial domain, resulting in a noticeable decline in model performance. In conclusion, the spatial view and feature view play crucial roles in STMIGCL. Each view characterizes the data from different dimensions, complementing each other and jointly enhancing the model's ability to understand and represent the data. By organically integrating these two views into the model's training process, STMIGCL can more comprehensively capture the features and structural information in spatial transcriptomics data, thereby significantly improving the model's performance and generalization capability. Finally, the experimental results of w/o‐A demonstrate that the attention mechanism is indispensable in the model training process. The attention mechanism allows the model to dynamically adjust its focus on different parts of the input data based on their importance, enabling a better understanding of the context. This dynamic allocation characteristic not only enhances the model's ability to process information during multi‐view fusion but also effectively improves its adaptability and generalization capability for complex data. Particularly in scenarios involving multi‐view collaboration, the attention mechanism can highlight the role of key information while mitigating the interference of less relevant information, thereby further optimizing the model's performance. In addition, to make the comparison between STMIGCL and its variants more intuitive, this study further visualized the clustering results of slice #151 507 from the DLPFC dataset, as shown in Figure 6D. The results reveal that, compared to the other two variants, w/o‐F produces more accurate clustering results overall. However, its delineation of Layer 2 to Layer 4 is less precise. For the variant using only feature information, the clustering results appear more chaotic, further highlighting the importance and indispensability of spatial coordinates in spatial domain recognition tasks. As for the w/o‐A variant, although it can effectively distinguish different layers, its performance in boundary processing is inadequate, with less smooth boundaries and the presence of many discrete points.

To further validate the critical role of each component in STMIGCL during the training process, we conducted a comparative analysis of STMIGCL and its variants with several state‐of‐the‐art methods on the DLPFC dataset. Specifically, these methods include Spatial‐MGCN (which integrates multi‐view learning and attention mechanisms but does not employ contrastive learning), GraphST (primarily reliant on contrastive learning but lacks multi‐view learning and attention mechanisms), STAGATE (focused on attention mechanisms without incorporating multi‐view learning and contrastive learning), and CCST (focused on contrastive learning and similarly lacks multi‐view learning and attention mechanisms). The bar chart in Figure 6C intuitively displays the average ARI and NMI values for each method, providing a clear performance comparison. The analysis results reveal that methods solely relying on contrastive learning (such as GraphST, CCST, and variants w/o‐F, w/o‐S) or attention mechanisms (STAGATE and variant w/o‐A) did not achieve optimal results. Spatial‐MGCN, by combining multi‐view learning and attention mechanisms, achieved relatively impressive average ARI and NMI values, highlighting that the combination of multi‐view learning and attention mechanisms can effectively enhance clustering performance. STMIGCL further optimizes the clustering performance of the model by incorporating contrastive learning. Therefore, the method proposed in this study effectively integrates various components to fully leverage the advantages of multi‐view learning and contrastive learning. It achieves optimal performance across all evaluation metrics, demonstrating its powerful capability in spatial transcriptomics data analysis and providing a robust tool for uncovering the latent information within spatial transcriptomics data.

In summary, the ablation study further validates and highlights the importance of incorporating spatial views, feature views, and attention mechanisms into STMIGCL. The effective integration of these components enables the model to achieve more accurate spatial clustering. Additionally, the comparative analysis with existing methods further underscores the advantages of STMIGCL. By combining multi‐view learning, contrastive learning, and attention mechanisms, STMIGCL not only provides a more comprehensive capture and representation of data features but also achieves outstanding performance across all evaluation metrics, fully demonstrating its potential and superior capability in spatial transcriptomics data analysis.

## 3 Discussion

Making full use of the gene expression profiles and spatial location information in spatial transcriptomics data to accurately identify spatial domains is crucial for researchers to gain a deeper understanding of tissue structure and biological functions. This study introduces STMIGCL, a multi‐view graph convolutional network framework leveraging implicit contrastive learning to explore latent information comprehensively. STMIGCL constructs multiple neighbor graphs using gene expression and spatial location data, each defined by distinct similarity measures. Then, the multi‐view graph convolutional network encoder is employed to derive low‐dimensional representations for different views, while the implicit contrastive learning approach is utilized to further enrich the latent representations of each view. Subsequently, an attention mechanism is utilized integrate the representations from diverse views in an adaptive manner, assigning different attention scores to different views to better capture their importance and obtain the spot representations of the whole model. The study presents a model that effectively utilizes the consistency information between different graph structures through multi‐view learning. By further enhancing the latent representations of spot through contrastive learning, thereby imbuing the learned representations with greater informativeness and discriminability, ultimately enhancing clustering performance.

To validate the effectiveness of STMIGCL, this study tested it on five datasets platforms for validation. The outcomes of the experiments demonstrate that the proposed approach achieves competitive performance compared to baseline methods. First, STMIGCL can fully explore the real potential adjacency relationships between spots through the Multi‐View Graph Convolutional Network encoder, and has stronger robustness to potential noise in the original data. By further using the implicit contrastive learning method, the obtained latent representations are made more discriminative and better preserve the underlying information of the original data. Second, by incorporating an attention mechanism, it can adaptively aggregate representations from different views. The results obtained in this study consistently indicate that STMIGCL effectively identifies spatial domains exhibiting consistent gene expression and histological characteristics. Furthermore, this method not only possesses the capability to identify SVGs with abundant expression patterns in established fields, but also, compared to the genes detected by conST and SpaGCN, these SVGs exhibit clearer spatial expression patterns and more profound biological interpretations. To comprehensively evaluate the robustness and generalization capability of our model, we conducted additional experiments on datasets generated by the osmFISH sequencing technique from the mouse somatosensory cortex, as well as on spatial ATAC‐seq data from mouse embryonic brains at E15.5. The results of these experiments are presented in Figures S6 and S7 (Supporting Information). Compared to baseline methods, STMIGCL demonstrated superior clustering performance, strongly confirming the effectiveness of our approach in handling datasets generated by different technical platforms.

As spatial transcriptomics data becomes increasingly accessible, it is anticipated that the generation of large‐scale datasets suitable for analysis will also become possible in the near future. However, the computational method of contrastive learning using Multi‐View Graph Convolutional Networks is memory‐intensive, posing challenges for their application on large‐scale ST datasets. Accordingly, to boost the scalability of our model, we might explore techniques like mini‐batch processing, parallelism, or distributed computing systems, thereby enhancing the model's efficiency. In addition, given the relative ease of acquiring histopathological images, there is potential to pretrain a large model for feature extraction from image data and incorporate it into the training process to enhance performance.

## 4 Experimental Section

### Data Description

To assess the efficacy of the proposed method, this study applies STMIGCL to five spatial transcriptomics datasets obtained by various technologies and platforms, encompassing 10x Visium, Stereo‐seq,^[^ ^42^ ^]^ and STARmap.^[^ ^10^ ^]^ Specifically, the first dataset was downloaded from the human dorsolateral prefrontal cortex (DLPFC),^[^ ^31^ ^]^ and was obtained utilizing the 10x Visium technology, comprising 12 tissue sections. The range of spots per section varies between 3460 and 4789, encompassing data from 33538 genes in total. To better analyze this data, each section underwent manual annotation, accurately dividing them into regions consisting of 5 to 7 areas. The second dataset comes from the SEDR platform, a human breast cancer dataset^[^ ^36^ ^]^ utilizing the 10x Visium technology obtained. This dataset was extensively analyzed and annotated by Xu et al.^[^ ^26^ ^]^ drawing upon H&E staining and pathological characteristics of human breast cancer samples. They not only segmented the dataset into 20 different regions but also classified them into four main types based on morphological features: ductal carcinoma in situ/lobular carcinoma in situ (DCIS/LCIS), invasive ductal carcinoma (IDC), healthy regions (Health), and low malignant tumor margins (Tumor edge). The third dataset comprised a collection of mouse olfactory bulb data generated through the utilization of Stereo‐seq methodology, comprising 19109 spots and 14376 genes. After processing with SEDR, the dataset was labeled as rostral migratory stream (RMS), granular cell layer (GCL), internal plexiform layer (IPL), mitral cell layer (MCL), external plexiform layer (EPL), glomerular layer (GL), and olfactory nerve layer (ONL). The fourth dataset was downloaded from Stereo‐seq data obtained from E9.5 mouse embryos. It comprises 5913 spots covering 25568 genes. Annotations of the tissue domains for these data were extracted from relevant original studies, providing a reliable foundation for further interpretation and analysis. The final dataset consists of the mouse visual cortex dataset acquired through STARmap, which includes the visual cortex and six neocortical areas from the hippocampus to the corpus callosum. A total of 1020 genes were detected in 1207 cells including non‐neuronal cells, excitatory and inhibitory neurons. Please refer to the Table S1 (Supporting Information) for detailed information on all datasets.

### Data Preprocessing

To mitigate technical noise within the dataset, a series of preprocessing procedures were executed. Initially, spots beyond the primary organizational areas were eliminated and discarded genes exhibiting expression in fewer than three points or cells. Following this, a logarithmic transformation was applied to the raw gene expression data using the SCANPY toolkit to better accommodate the data distribution characteristics during analysis. Subsequent normalization based on library size was conducted to alleviate inter‐sample technical discrepancies. Ultimately, the top 3000 highly variable genes were extracted from the preprocessed dataset and utilized them as input for the STMIGCL, facilitating further model training and analysis.

### Graph Construction

Spatial transcriptomics boasts its strength in providing spatial information about tissue cells, aiding in the identification of similar cell states. These states were also spatially co‐localized, thereby dividing tissue substructures. To fully exploit the information in spatial transcriptomics data, this study adopts two similarity measures commonly used in the majority of existing graph‐based spatial transcriptomics clustering methods^[^ ^20^, ^30^ ^]^ to construct the adjacency matrix.

First, to fully capitalize on spatial information, a spatial graph was established using positional coordinates *G* ^(1)^ = (*V*, *E*), where *V* represents the spot set, *E* represents the edges connecting various spots, and its adjacency matrix is expressed as *A* ^(1)^ ∈ *R* ^*N* × *N*^, where *N* represents the spots count. If spot *j* ∈ *V* is adjacent to spot *i* ∈ *V*, $A_{ij}^{\left(1\right)}=1$ was set, otherwise, 0 was set. Therefore, the determination of neighbors for a particular spot was facilitated by calculating the Euclidean distance from its spatial coordinates, which defines the proximity of that spot to other spots. The Euclidean distance serves as an effective metric for measuring the spatial separation between two points across multiple dimensions, and its formal expression is:

(1) $$A_{ij}^{\left(1\right)}=\left\{\begin{array}{c} 1,\;if\;S_{ij}<r\\ 0,\;otherwise \end{array}\right.$$

where *S_ij_* represents the Euclidean distance, and *r* is the predefined radius (default *r* = 700).

Second, to uncover the latent structure of gene expression, the similarity of gene expression was further calculated by cosine distance. Cosine similarity measures the degree of proximity between two points aligned in the same direction, which was not affected by the feature dimension and scale. Specifically, mainly *k*‐nearest neighbor graph was constructed utilizing gene expression matrix *G* ^(2)^ = (*V*, *E*) and the *k* is default to 20. The cosine similarity was computed as:

(2) $$sim\left(x_{i},x_{j}\right)=\frac{x_{i}\cdot x_{j}}{\left|x_{i}\right|\left|x_{j}\right|}$$

where *x_i_* and *x_j_* respectively represent the features of spot *i* and spot *j*.

### Multi‐View Graph Contrastive Learning—Convolutional Network

GCN, a standout graph neural network, exhibits remarkable abilities in directly manipulating graph data and thoroughly delving into graph structural information. It gathers data from neighboring nodes, captures inter‐node dependencies, and produces informative embeddings. Considering that the current spatial transcriptome technology was still insufficient, it might cause the model to be susceptible to noise in the original data during the training phase. In order to make full use of the advantages of GCN in capturing graph structure data, in addition to constructing graphs based on spatial coordinate information, full use of the similarity between gene expression should be made. The graph constructed by expressing similarity could provide more information sources for GCN and further explore the potential patterns and relationships of spatial transcriptome data. Through the multi‐view learning strategy, not only the data could analyzed from multiple perspectives, but also make full use of the complementary advantages between different views, so as to improve the overall performance of the model. Therefore, to accurately extract crucial information from gene expression profiles and spatial structures, the adjacency matrix constructed was harnessed and apply a multi‐view GCN encoder for performing convolution operations on both feature maps and spatial maps. The encoder integrates both graph structural information and node feature attributes, iteratively aggregating representations from neighboring nodes to extract latent features from multiple perspectives.

In this study, to merge gene expression profiles with spatial location data, convolutional operations were initially conducted on each neighboring graph, with the goal of learning a mapping function *f*(*A*, *X*, θ) → *Z*. This mapping function transforms gene expression *X* into latent feature representations *Z*, leveraging the adjacency *A*, where θ is the model parameter. Specifically, the *l*‐th encoder layer could be expressed as:

(3) $$Z_{l}^{\left(i\right)}=\sigma \left(A^{\left(i\right)}Z_{l-1}^{\left(i\right)}W_{l-1}\right)$$

where *W*_*l* − 1_ represents the adjustable weight parameters for the *l*‐th layer, $A=D^{-\frac{1}{2}}AD^{-\frac{1}{2}}$ denotes the normalized adjacency matrix, and σ(·) represents the activation function (ReLU). The input and output of the *l*‐th layer was represented as $Z_{l-1}^{\left(i\right)},Z_{l}^{\left(i\right)}$, where $Z_{0}^{\left(i\right)}=X$, and *Z* ^(*i*)^ serves as the encoder's ultimate output, where row *i*‐th *z_i_* represents the latent representation of spot *i*.

### Multi‐View Graph Contrastive Learning—Graph Contrastive Learning with Implicit Enhancement

To enhance the informativeness and discriminativeness of the obtained representations *Z* ^(*i*)^, furthera graph contrastive learning strategy was employed^[^ ^49^ ^]^ with implicit enhancement. At present, the majority of graph contrastive learning approaches employ augmentation strategies rooted in random perturbations,^[^ ^50^, ^51^ ^]^ including randomly adding or deleting edges or nodes in the graph. These augmentation strategies were premised on the assumption that subtle, random alterations would not materially affect the semantic characteristics of the original graph.^[^ ^52^ ^]^ However, this hypothesis encompasses two pivotal concerns. First, manipulating edges or nodes in the graph might potentially distort the original data. Second, the perturbation level of such methods was often considered as a highly sensitive hyperparameter. To enhance the effectiveness of contrastive learning, an enhancement strategy was adopted based on latent space. Unlike simple random enhancement‐based methods, representations learned through deep learning capture higher‐level, more abstract features,^[^ ^53^ ^]^ which better preserve the critical information within the data. VGAE could not only combine graph structure and feature information for deep representation learning, but also model node embeddings as probability distributions. It was precisely because of the powerful probabilistic modeling ability of VGAE that the probability distribution obtained by VGAE was used as a contrast source and contrast enhancement sampled from the probability distribution could effectively avoid the limitations of random enhancement‐based methods. However, while VGAE had reconstruction capabilities, its latent distributions often overlap, making it difficult to separate the latent embeddings used for downstream tasks. Therefore, combining VGAE with contrastive learning could effectively alleviate this problem. Contrastive learning aims to separate embeddings in the representation space, and contrastive enhancement through the latent distribution generated by VGAE in turn further improves the effectiveness of contrastive learning. Therefore, combining contrastive learning based on implicit enhancement methods on the basis of multi‐view learning could not only extract richer information from the original data, but also significantly improve the discriminability and accuracy of the obtained latent representations.

Specifically, the objective was to train a VGAE that was proficient in reconstructing the graph topology, thereby establishing a solid basis for implicit contrastive learning. Adopting GCN as encoder, it was assumed that the latent representation $a_{n}\in R^{D}$ of the last layer of the node *n* follows Gaussian distribution $N(\mu_{n},diag\left(\sigma_{n}^{2}\right))$, where μ_*n*_ and σ_*n*_ were parameterized as:

(4) $$H_{\mu }^{\left(l\right)}=ReLU\left({\tilde{D}}^{-\frac{1}{2}}\tilde{A}{\tilde{D}}^{-\frac{1}{2}}H_{\mu }^{\left(l-1\right)}W_{\mu }^{\left(l\right)}\right)$$

(5) $$H_{\sigma }^{\left(l\right)}=ReLU\left({\tilde{D}}^{-\frac{1}{2}}\tilde{A}{\tilde{D}}^{-\frac{1}{2}}H_{\sigma }^{\left(l-1\right)}W_{\sigma }^{\left(l\right)}\right)$$

where ${\tilde{D}}_{ii}=\sum_{j=1}^{N_{spot}}{\tilde{A}}_{ij}$ represents the degree matrix of $\tilde{A}$ with $\tilde{A}=A+I$, the encoding network for both the mean and variance utilizes identical weights at its initial layer (i.e., $W_{\mu }^{\left(0\right)}=W_{\sigma }^{\left(0\right)}$), employing *ReLU*(·) as the activation function, *l = 0* where $H_{\mu }^{\left(0\right)}=H_{\sigma }^{\left(0\right)}=X$. The loss function comprises two main components: the reconstruction loss, which quantifies the similarity between the generated graph and the original one, and the Kullback‐Leibler (KL) divergence, which measures the dissimilarity between the distribution of node representation vectors and the normal distribution, represented as:

(6) $$L_{VGAE}=E_{q(H|X,A)}\left[\log p\left(A|H\right)\right]-KL\left[q\left(H|X,A\right)||p\left(H\right)\right]$$

where the initial component represents the probability of reconstruction achieved through an inner‐product decoder $p\left(A|H\right)=\Pi_{i=1}^{N}\Pi_{j=1}^{N}\sigma \left({a_{i}}^{T}a_{j}\right)$ (σ(·) serves as the logistic sigmoid function). The subsequent term serves to regularizes the Kullback‐Leibler divergence, comparing the variational distribution $q\left(H|X,A\right)=\Pi_{n=1}^{N}q\left(a_{n}|X,A\right)$ with a pre‐selected prior $p\left(H\right)=\Pi_{n=1}^{N}p\left(a_{n}\right)=\Pi_{n=1}^{N}N\left(a_{n}|0,I\right)$.

Drawing inspiration from the InfoNCE goal in contrastive learning, *M* samplings were performed from the latent distribution *N*(μ_*n*_,Σ_*n*_), with $a_{n}^{m}$ representing the *m*‐th augmented for node *n*. For each augmentation instance, $\left(a_{n}^{m},z_{n}\right)$ was considered as the positive sample, and ${\left\{\left(a_{n}^{m},z_{n^{\prime }}\right)\right\}}_{\left[n^{\prime }\neq n\right]}$ as the negative sample. The contrastive loss was then expressed as follows:

(7) $$L_{CL}=\frac{1}{N}\sum_{n=1}^{N}\frac{1}{M}\sum_{m=1}^{M}-\log\frac{\exp(z_{n}^{T}a_{n}^{m}/\tau )}{\sum_{n^{\prime }=1}^{N}\exp(z_{n^{\prime }}^{T}a_{n}^{m}/\tau )}$$

where the inner‐product scaled was employed by a temperature hyper‐parameter τ as the similarity metric.

### Attention Mechanism

The significance of gene expression and spatial information varies considerably in spatial domain recognition tasks. Utilizing aforementioned operations on spatial gene expression data, which were distinguished by distinct adjacency matrices, enables the derivation of embeddings capable of encapsulating points with varied graph structures. To seamlessly integrate these diverse embeddings into a cohesive representation, reflecting their individual significance, STMIGCL incorporates the following attention mechanism.^[^ ^54^ ^]^

(8) $$\left(\alpha^{\left(1\right)},\alpha^{\left(2\right)}\right)=att\left(Z^{\left(1\right)},Z^{\left(2\right)}\right)$$

where α^(1)^ and α^(2)^ represent the attention coefficients, respectively. *att*(·) represents the attention mechanism. Specifically, the point embeddings undergo a linear transformation to derive distinct attention values, i.e. $v_{j}^{i}=qz$, where *q* serving as the common attention vector and $z_{j}^{i}$ represents the *j*‐th spot of the *i*‐th embedding. Following this, softmax function was applied to normalize the attention values, ensuring comparability among coefficients for different points. The ultimate embedding for a given spot was determined by the following formula:

(9) $$\alpha_{i}^{\left(1\right)}=softmax\left(v_{i}^{\left(1\right)}\right)=\frac{\exp(v_{i}^{\left(1\right)})}{\sum_{j=1}^{2}\exp(v_{i}^{\left(j\right)})}$$

Similarly, it have $\alpha_{i}^{\left(2\right)}=softmax\left(v_{i}^{\left(2\right)}\right)$. Let $\alpha^{\left(1\right)}=\left[\alpha_{i}^{\left(1\right)}\right]$, $\alpha^{\left(2\right)}=\left[\alpha_{i}^{\left(2\right)}\right]$ and α^(1)^ = *diag*(α^(1)^), α^(2)^ = *diag*(α^(2)^), the combined embedding *Z* could be derived by merging *Z* ^(1)^ and *Z* ^(2)^, i.e. *Z* = α^(1)^ · *Z* ^(1)^ + α^(2)^ · *Z* ^(2)^

### Spatial Domain Identification by Clustering

To enhance the precision of spatial domain recognition, iterative unsupervised deep embedding clustering framework was utilized that assigns each point to its specific domain. Initially, the k‐means algorithm was applied to initialize the cluster centroids μ_*j*_ by leveraging the final embedding *Z*, where μ_*j*_(*j* = 1, …, *C*) denotes the centroids of the *j*‐th cluster, and *C* represents the total count of distinct cell types. To enhance clustering efficiency, a two‐phase alternating approach was employed, the first phase involves computing the similarity between spot embeddings *z_j_* and μ_*j*_ using the Student's t‐distribution:

(10) $$q_{ij}=\frac{{\left(1+{\left\|z_{i}-\mu_{j}\right\|}^{2}\right)}^{-1}}{\sum_{j^{\prime }}{\left(1+{\left\|z_{i}-\mu_{j^{\prime }}\right\|}^{2}\right)}^{-1}}$$

where *q_ij_* represents the likelihood of spot *i* being allocated to cluster *j*. In the second phase, iteratively refining the clustering process involves using the target distribution *p_ij_* to enhance the current high‐confidence assignments, which was derived as described below:

(11) $$p_{ij}=\frac{q_{ij}^{2}/\sum_{i}q_{ij}}{\sum_{j^{\prime }}q_{ij^{\prime }}^{2}/\sum_{i}q_{ij^{\prime }}}$$

After deriving the soft distribution *q_ij_* and the auxiliary target distribution *p_ij_* through the aforementioned two‐phase process, stochastic gradient descent with momentum was used to minimize the Kullback‐Leibler (KL) divergence loss between two distributions to simultaneously optimize the network parameters and cluster centers:

(12) $$L_{KL}=KL\left(P||Q\right)=\sum_{i}\sum_{j}p_{ij}\log\frac{p_{ij}}{q_{ij}}$$

Upon completion of training, the cluster assignment for spot *i* is determined by utilizing $\arg\max_{j}q_{ij}$.

### Overall Loss Function

During the training process, the KL divergence loss and the implicit contrastive loss was jointly optimized. The ultimate training objective of STMIGCL was defined as:

(13) $$L=\alpha L_{KL}+\beta L_{CL}$$

where α and β are weight factors that balance the KL divergence loss and the implicit contrastive loss.

### Evaluation Metrics—Clustering Performance

The clustering results were assessed using two widely employed metrics, namely the Adjusted Rand Index^[^ ^55^ ^]^ and Normalized Mutual Information.

(14) $$ARI=\frac{\sum_{ij}\left(\begin{array}{c} n_{ij}\\ 2 \end{array}\right)-\frac{\left[\sum_{i}\left(\begin{array}{c} a_{i}\\ 2 \end{array}\right)\sum_{j}\left(\begin{array}{c} b_{j}\\ 2 \end{array}\right)\right]}{\left(\begin{array}{c} n\\ 2 \end{array}\right)}}{\frac{1}{2}\left[\sum_{i}\left(\begin{array}{c} a_{i}\\ 2 \end{array}\right)+\sum_{j}\left(\begin{array}{c} b_{j}\\ 2 \end{array}\right)\right]-\frac{\left[\sum_{i}\left(\begin{array}{c} a_{i}\\ 2 \end{array}\right)\sum_{j}\left(\begin{array}{c} b_{j}\\ 2 \end{array}\right)\right]}{\left(\begin{array}{c} n\\ 2 \end{array}\right)}}$$

where *a_i_* and *b_j_* represent the counts of samples present within the *i*‐th prediction cluster and *j*‐th actual cluster, respectively, and *n_ij_* signifies the quantity of samples that overlap between the *i*‐th predicted cluster and the *j*‐th actual cluster.

(15) $$NMI\left(Y,C\right)=\frac{2\times [H(Y)-H(Y|C)]}{\left[H(Y)+H(C)\right]}$$

where *C* denotes the predicted cluster, and *Y* represents the actual cluster. The entropy was computed using the function designated as *H*(·).

ARI evaluates the similarity between clustering outcomes and their corresponding true labels. Conversely, NMI quantifies the mutual information between labels.

### Evaluation Metrics—Spatial Autocorrelation

The expression levels of genes across different locations might not be mutually independent. Specifically, genes situated in nearby regions tend to exhibit numerically closer expression levels than those situated further apart. This phenomenon, where gene expression demonstrates similarity due to spatial variation, was termed spatial autocorrelation, reflecting the correlation of a variable with itself across distinct spatial points. To investigate whether the detected SVGs display a structured spatial expression pattern, the Moran's I^[^ ^56^ ^]^ index was employed as a metric to quantify the extent of spatial autocorrelation in gene expression. Utilizing the following formula to compute its Moran's I value:

(16) $$I=\frac{N}{W}\frac{\sum_{i}\sum_{j}\left[w_{ij}\left(x_{i}-\bar{x}\right)\left(x_{j}-\bar{x}\right)\right]}{\sum_{i}{\left(x_{i}-\bar{x}\right)}^{2}}$$

where *x_i_* and *x_j_* denote the expression levels of gene at spots *i* and *j* respectively, $\bar{x}$ signifies the average gene expression level across all spots. Furthermore, *w_ij_*, which was calculated based on the 2D spatial coordinates of spots *i* and *j*, denotes the spatial weight between these two spots. *W* represents the summation of *w_ij_*, providing a measure of the relative influence of spatial proximity on gene expression levels

### Parameter Setting

The whole model was implemented utilizing Python 3.9 and PyTorch 2.0.0. In this experiment, all views use single graph convolution layer. To optimize the model, the Adam optimizer was utilized, setting a learning rate of 0.003 and weight decay of 0.001. For detailed parameter information about the model, please refer to Table S4 (Supporting Information). Consistency was maintained with prior research by strictly following the default parameters proposed in the original papers for all baseline methods. This approach ensures a fair evaluation of the performance of various baseline methods. All experiments were performed on high‐performance CPU/CUDA server (CPU: AMD Radeon (TM) Graphics, GPU: NVIDIA‐SMI 510.73.05).

### Algorithm

This study presents a multi‐view graph convolutional network framework based on implicit contrastive learning, STMIGCL, to comprehensively exploit the latent information in spatial transcriptomics data. The following pseudo‐code describes the steps of the algorithm in detail (Algorithm 1).

Algorithm 1 **STMIGCL**

**Table:** 

**Input**: Input data: *X*; Graph: *G* ^(1)^ and *G* ^(2)^; Maximum iterations: *MaxIter*; Sample size: *b*; Target distribution update interval: *T*; Stopping threshold: δ.	
**Parameter**: Φ^(1)^, Φ^(2)^ in *VGAE*(·); Θ in GNN backbone *F*(·) and attention layer *att*(·).	
1:	Input *X*, *G* ^(1)^ and *G* ^(2)^ into the model.
2:	Initialize μ by k‐means.
3:	for *iter* ∈ {0, 1, …, *MaxIter*} do
4:	Generate $\mu_{n}^{\left(1\right)}$, $\sigma_{n}^{\left(1\right)}$ and $\mu_{n}^{\left(2\right)}$, $\sigma_{n}^{\left(2\right)}$ from *VGAE*(·).
5:	Update Φ^(1)^, Φ^(2)^ by *L_VGAE_* in Equation (6) respectively.
6:	Generate $z_{n}^{\left(1\right)}$, $z_{n}^{\left(2\right)}$ from *F*(·).
7:	Randomly sample a batch of size *b* from ${\left\{z_{n}^{\left(1\right)}\right\}}_{n=1}^{N}$, ${\left\{z_{n}^{\left(2\right)}\right\}}_{n=1}^{N}$ respectively.
8:	Compute $L_{CL}^{\left(1\right)}$, $L_{CL}^{\left(2\right)}$ by Equation (7), and $L_{CL}=L_{CL}^{\left(1\right)}+L_{CL}^{\left(2\right)}$.
9:	Generate *z_n_* from *att*(·).
10:	**if** *iter*%*T* = = 0 **then**
11:	Update *P* using Equations (10), (11) and *z_n_*.
12:	Compute label assignments *s* by $\arg\max_{j}q_{ij}$.
13:	Save last label assignment: *s_old_* = *s*.
14:	**if** *sum*(*s_old_* ≠ *s*)/*n* < δ **then**
15:	Stop training.
16:	Compute *L_KL_* by Equation (12).
17:	Update Θ by *L* in Equation (13).
18:	**end for**
19:	**return** Embedding **Z** and label assignment **S**.

## Conflict of Interest

The authors declare no conflict of interest.

## Author Contributions

B.Y. and S.R. conceived this study. B.Y. and X.G. initiated the study. S.R. and X.Y.L. implemented the STMIGCL method. F.R.L. and J.L. completed the data analysis. S.R. and X.Y.L. wrote the manuscript under supervision of B.Y. and X.G. All authors are involved in discussion and finalization of the manuscript.

## Supporting information



Supporting Information

## Data Availability

The complete codebase utilized in this study is openly accessible at https://github.com/YuBinLab‐QUST/STMIGCL/. The datasets employed in our research are publicly accessible and downloadable. Specifically, (1) the DLPFC dataset is accessible through the spatialLIBD package at http://spatial.libd.org/spatialLIBD; (2) 10x Visium spatial transcriptomics dataset of human breast cancer https://github.com/JinmiaoChenLab/SEDR_analyses/tree/master/data; (3) the processed Stereo‐seq data from mouse olfactory bulb tissue is accessible on https://github.com/JinmiaoChenLab/SEDR_analyses; (4) the Stereo‐seq data acquired from mouse embryos at E9.5 can be downloaded from https://db.cngb.org/stomics/mosta/; (5) the mouse visual cortex STARmap data is accessible on https://www.starmapresources.com/data. (6) the osmFISH mouse somatosensory cortex data is accessible on http://linnarssonlab.org/osmFISH/availability/.
