# Supplementary material for: Exploring the Latent Information in Spatial Transcriptomics Data via Multi‐View Graph Convolutional Network Based on Implicit Contrastive Learning
Source: Adv Sci (Weinh). 2025 Apr 30;12(21):2413545. doi: 10.1002/advs.202413545 (PMC12140381; doi:10.1002/advs.202413545)
Supplement: Supplementary file 1 — Supporting Information [file ADVS-12-2413545-s001.pdf]

ADVANCED SCIENCE
Open Access

Supporting Information

for *Adv. Sci.*, DOI 10.1002/advs.202413545

Exploring the Latent Information in Spatial Transcriptomics Data via Multi-View Graph Convolutional Network Based on Implicit Contrastive Learning

Sheng Ren, Xingyu Liao, Farong Liu, Jie Li, Xin Gao and Bin Yu**

Supplementary materials:

Exploring the latent information in spatial transcriptomics data via multi-view graph convolutional network based on implicit contrastive learning

Sheng Ren[1], Xingyu Liao[2], Farong Liu[3], Jie Li[1], Xin Gao[4,*] and Bin Yu[1,5,*]

[1]School of Data Science, Qingdao University of Science and Technology, Qingdao 266061, China

[2]School of Computer Science, Northwestern Polytechnical University, Xi'an 710072, China

[3]College of Mathematics and Physics, Qingdao University of Science and Technology, Qingdao 266061, China

[4]Computational Bioscience Research Center (CBRC), Computer, Electrical and Mathematical Sciences and Engineering Division, King Abdullah University of Science and Technology (KAUST), Thuwal 23955, Saudi Arabia

[5]School of Artificial Intelligence and Data Science, University of Science and Technology of China, Hefei 230026, China

*All correspondence should be addressed to BY (yubin@qust.edu.cn) and XG (xin.gao@kaust.edu.sa).

Table of contents

1. Supplementary Notes

1.1 Introduction of baseline methods.

1.2 Detection of spatial variation genes.

2. Supplementary Figures

Figure S1. Comparison of spatial domains by clustering assignments via STMIGCL, Spatial-MGCN, PAST, GraphST, STAGATE, CCST, SEDR, SpaGCN, SCANPY, and manual annotation in all 12 sections of the DLPFC dataset.

Figure S2. UMAP visualization and PAGA graphs generated by STMIGCL, Spatial-MGCN, PAST, GraphST, STAGATE, SEDR, SpaGCN, and SCANPY embeddings respectively.

Figure S3. Statistical difference of ARI and NMI values between each pair of methods on the DLPFC dataset.

Figure S4. Differential expression analysis between cluster 0 (DCIS/LCIS region) and cluster 5 (DCIS/LCIS edge) in human breast cancer dataset.

Figure S5. The performance of our proposed method was influenced by changing the weights of losses.

Figure S6. Spatial domains detected on osmFISH Mouse Somatosensory Cortex (MSC) dataset.

Figure S7. Application of STMIGCL to the spatial ATAC-seq data.

3. Supplementary Tables

Table S1. Summary of the datasets used in our study.

Table S2. The quantitative evaluation results of STMIGCL and baseline methods on the Stereo-seq dataset of E9.5 mouse embryos.

Table S3. Performance variations on DLPFC dataset by using the three variants of STMIGCL to train the model.

Table S4. Setting of parameters for the STMIGCL.

1. Supplementary Notes

1.1 Introduction of baseline methods

This study compared STMIGCL with non-spatial clustering methods implemented through SCANPY[1], as well as six recently developed spatial clustering methods (including Spatial-MGCN[2], PAST[3], GraphST[4], STAGATE[5], CCST[6], BayesSpace[7], SpaGCN[8], and SEDR[9]).

SCANPY: SCANPY is an extensible toolkit for analyzing single-cell gene expression data, built in conjunction with anndata. Data preprocessing in SCANPY involves log normalization and selection of the top 3000 highly variable genes (HVGs). It calculates the top 30 principal components (PCs) and then constructs a nearest neighbor graph using the "scanpy.pp.neighbor()" function with default parameters. Finally, SCANPY uses the "scanpy.tl.louvain()" function to obtain clustering assignments. For the DLPFC dataset, the resolution parameter was manually adjusted to ensure that the number of clusters equals the true value.

Spatial-MGCN：Spatial-MGCN utilizes a multi view graph convolutional network with the attention mechanism to effectively model gene expression and spatial location. All experiments were performed using the parameters recommended in the original studies.

PAST：PAST proposes a self-attention framework built on priors, serving as a variational graph convolutional autoencoder tailored for ST data. All experiments were conducted using the parameters recommended in the vignette package.

GraphST：GraphST is a graph self-supervised contrastive learning method that combines graph neural networks with self-supervised contrastive learning. It learns informative and discriminative node representations by minimizing the embedding distance between spatially neighboring spots. All experiments were conducted using the parameters recommended in the vignette package.

STAGATE：STAGATE is a method based on a deep learning model that combines autoencoders with graph attention mechanisms to learn latent representations by modeling gene expression profiles and spatial positional information. All experiments were conducted using the parameters recommended in the vignette package.

CCST：CCST utilizes GCN to achieve unsupervised spatial clustering by integrating complex global spatial information from gene expression at individual points and spatial gene expression. Following the tutorial, this study applied CCST to spatial clustering tasks using default parameter

settings.

BayesSpace：BayesSpace employs a Bayesian model with a Markov random field, utilizing spatial and gene expression information to model clustering of spatial transcriptomic data. This study followed the BayesSpace analysis tutorial and used the following parameters: nrep=50,000, gamma=3, platform="Visium", and mode="normal".

SpaGCN：SpaGCN is a graph convolutional network method that integrates gene expression, spatial positional information, and histological images for spatial transcriptomics data analysis. Following the tutorial, this study applied SpaGCN to spatial clustering tasks using default parameter settings.

SEDR：SEDR utilizes a deep autoencoder network to learn latent representations of genes, while embedding spatial information through a variational graph autoencoder to generate unsupervised spatial embedding representations. All experiments were conducted using the parameters recommended in the vignette package.

1.2 Detection of spatial variation genes

In this study, we followed the same procedure as proposed in SpaGCN to detect genes using SVG based on the domains identified by STMIGCL. This method detects genes that are enriched in specific spatial domains by searching for neighboring points within a predefined radius around each point in the target domain. A domain is defined as neighboring if more than 50% of the points in it are neighbors. After obtaining the neighboring domains, the Wilcoxon rank-sum test is used to determine differentially expressed genes between the target domain and its neighboring domains. Genes with adjusted p-values <0.05 are selected as SVGs. Additionally, genes must meet three additional criteria: 'domain internal score' greater than 80%, 'input/output score ratio' exceeding 1, and 'input/output expression ratio' greater than 1.5, to ensure their substantial expression in the target domain.

2. Supplementary Figures

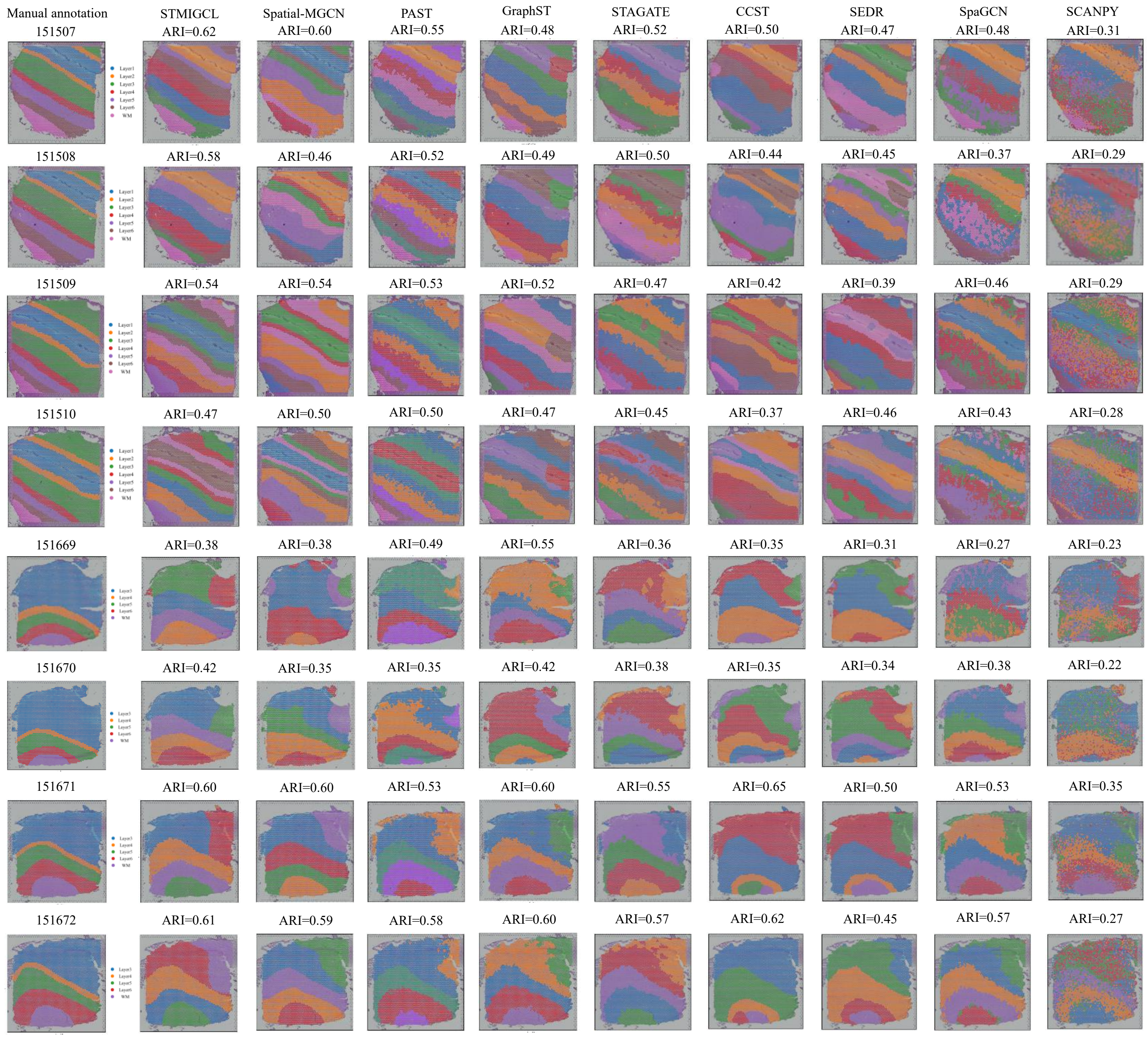

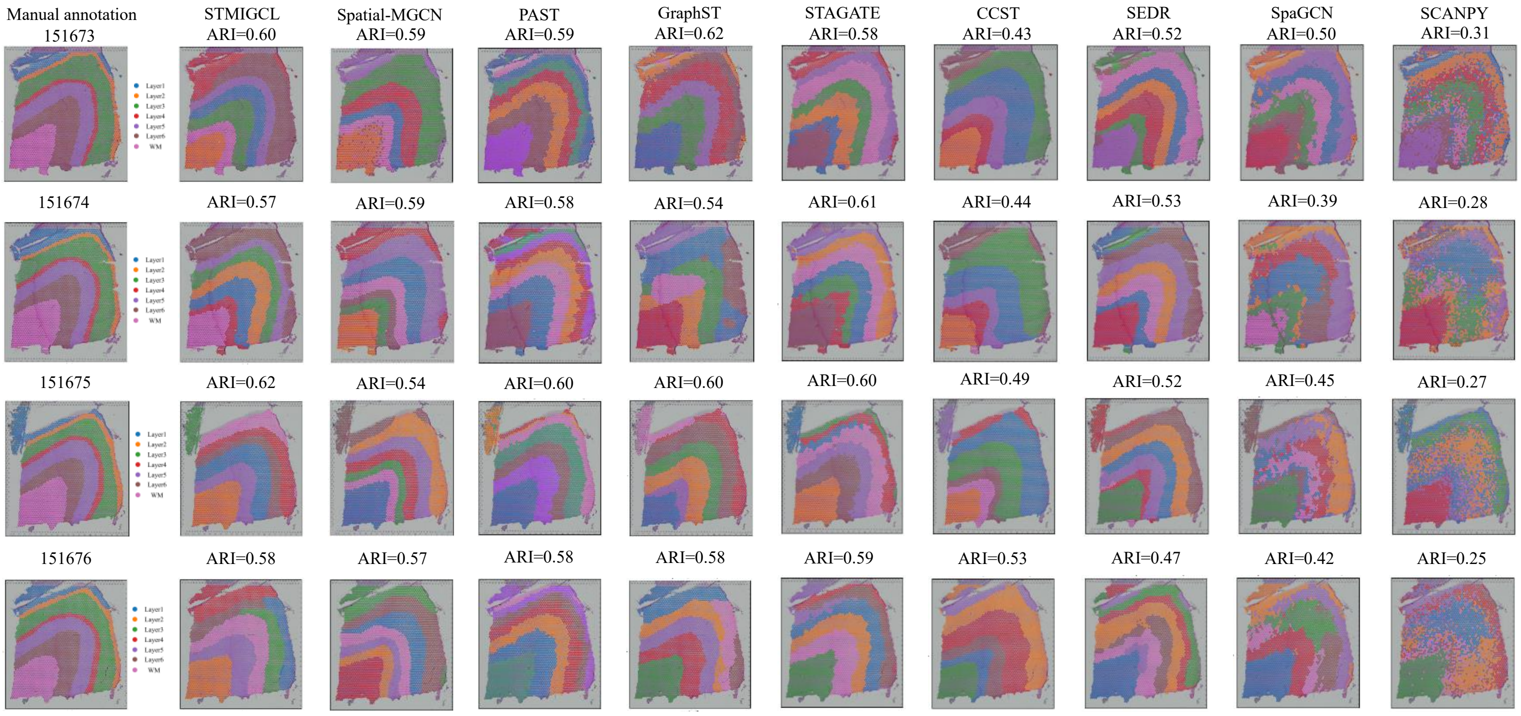

Figure S1. Comparison of spatial domains by clustering assignments via STMIGCL, Spatial-MGCN, PAST, GraphST, STAGATE, CCST, SEDR, SpaGCN, SCANPY, and manual annotation in all 12 sections of the DLPFC dataset.

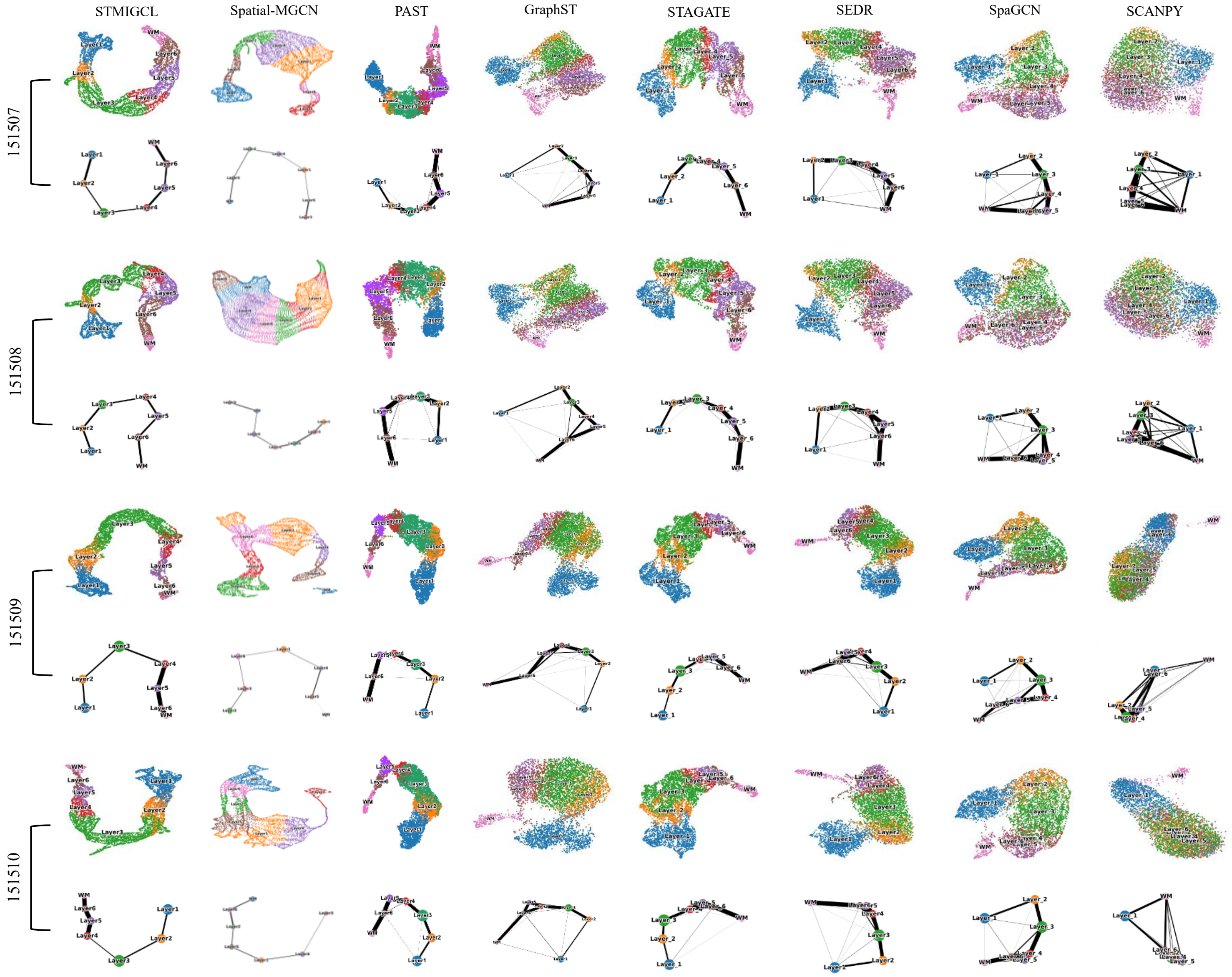
STMIGCL
Spatial-MGCN
PAST
GraphST
STAGATE
SEDR
SpaGCN
SCANPY
151507
151508
151509
151510

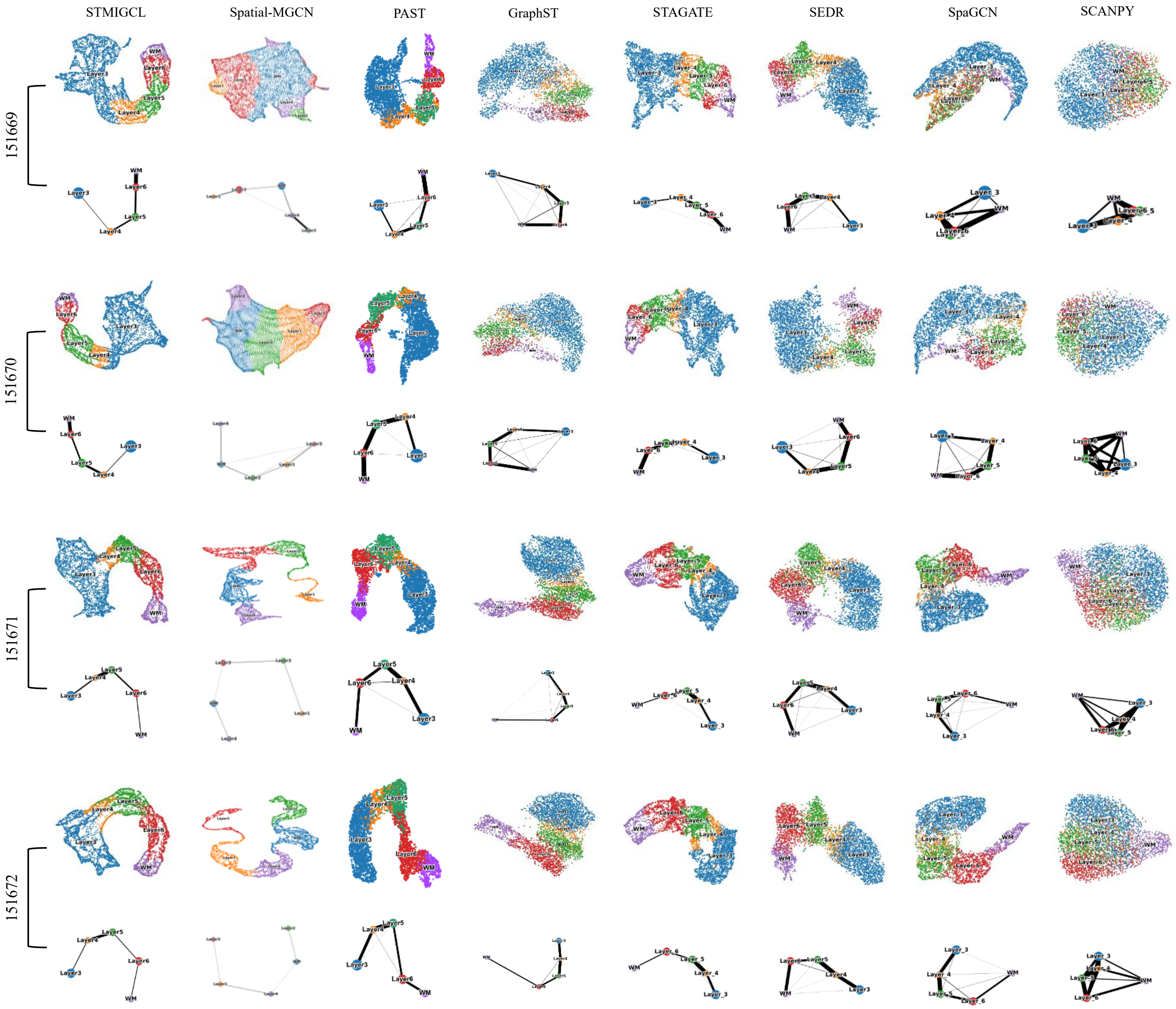
STMIGCL
Spatial-MGCN
PAST
GraphST
STAGATE
SEDR
SpaGCN
SCANPY
151669
151670
151671
151672

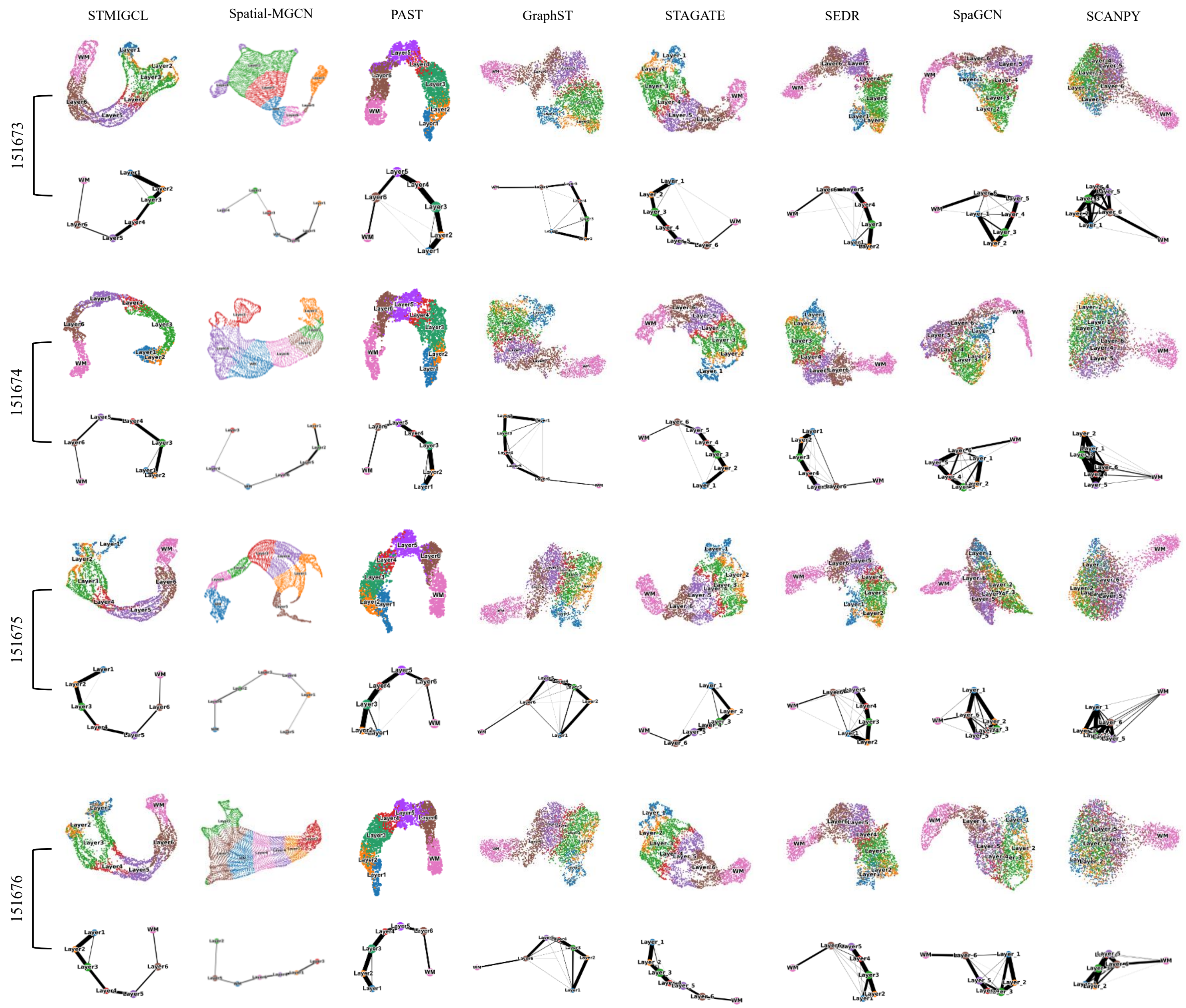

Figure S2. UMAP visualization and PAGA graphs generated by STMIGCL, Spatial-MGCN, PAST, GraphST, STAGATE, SEDR, SpaGCN, and SCANPY embeddings respectively.

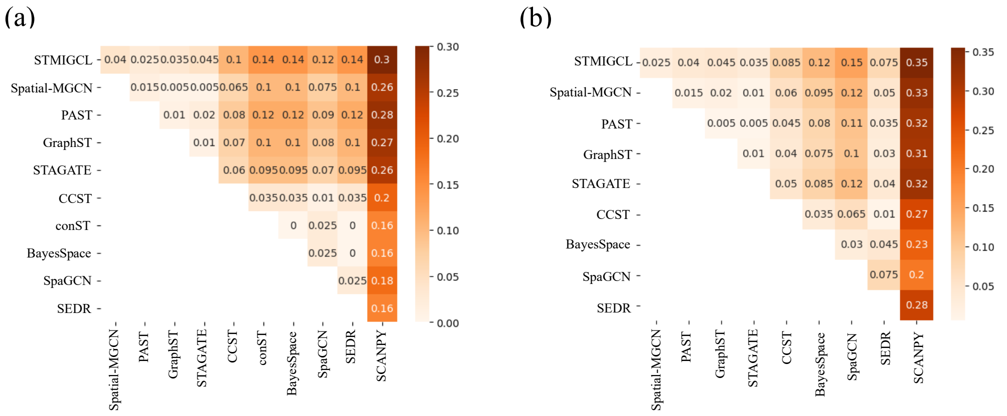

Figure S3. (a) Statistical difference of ARI values between each pair of methods on the DLPFC dataset. (b) Statistical difference of NMI values between each pair of methods on the DLPFC dataset.

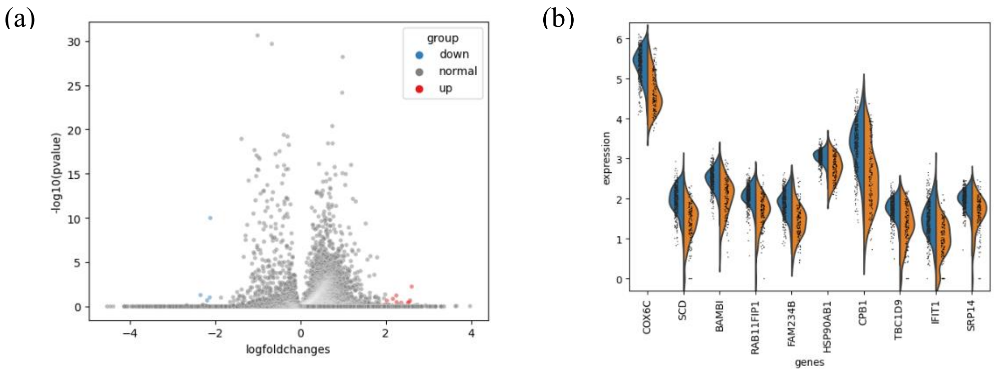

Figure S4. Differential expression analysis between cluster 0 (DCIS/LCIS region) and cluster 5 (DCIS/LCIS edge) in human breast cancer dataset. (a) Volcano plot of DEGs between cluster 0 and 5. (b) Differential expression of the top 10 genes between cluster 0 and 5.

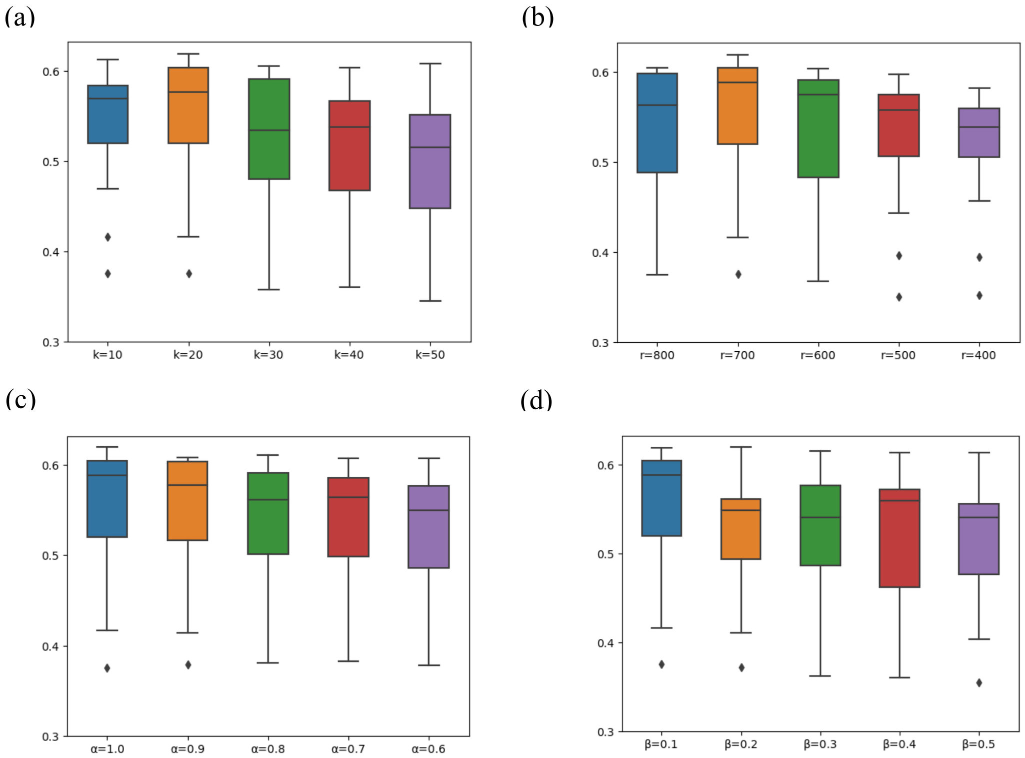

Figure S5. The performance of our proposed method was influenced by changing the weights of losses. (a) Feature graph parameter k. (b) Spatial graph parameter r. (c) Weight factor α. (d) Weight factor β.

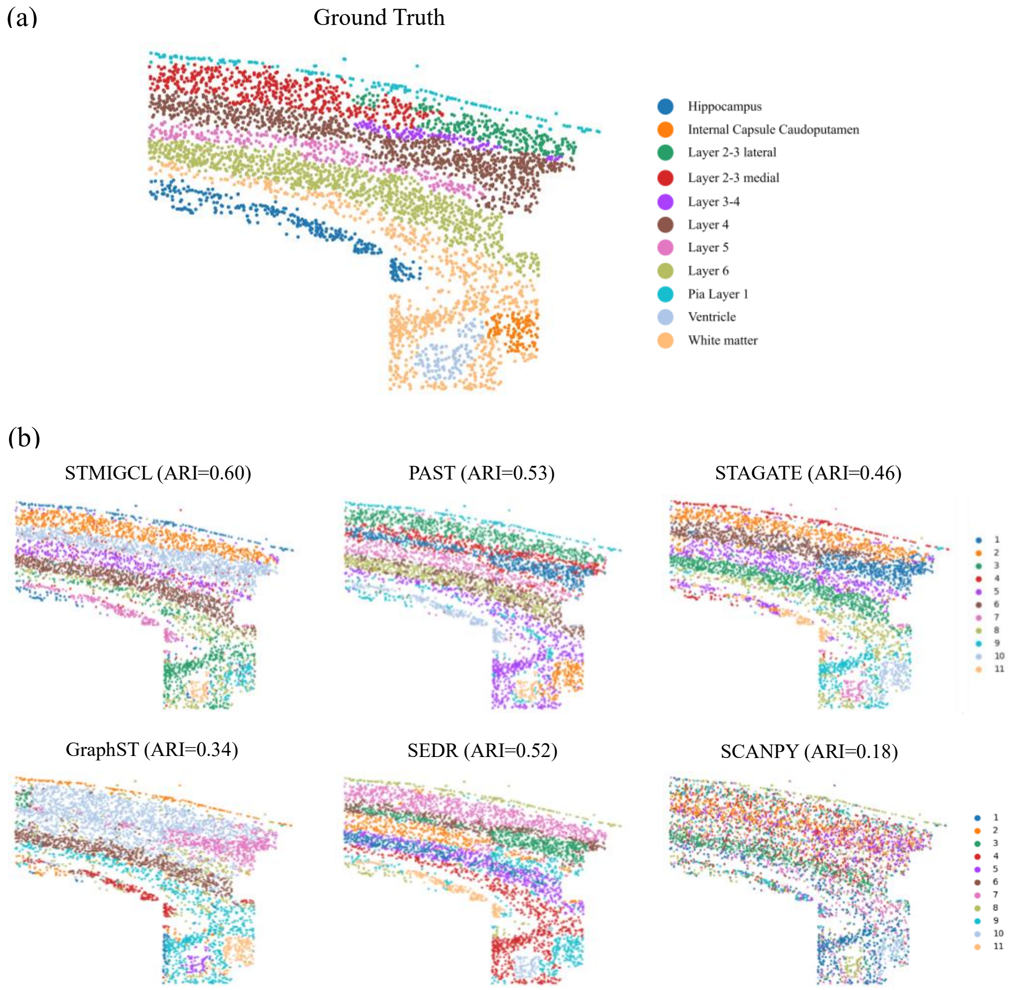

Figure S6. Spatial domains detected on osmFISH Mouse Somatosensory Cortex (MSC) dataset. (a) Layer structure of the tissue section from the original study. (b) Spatial domains identified by STMIGCL, PAST, STAGATE, GraphST, SEDR and SCANPY respectively.

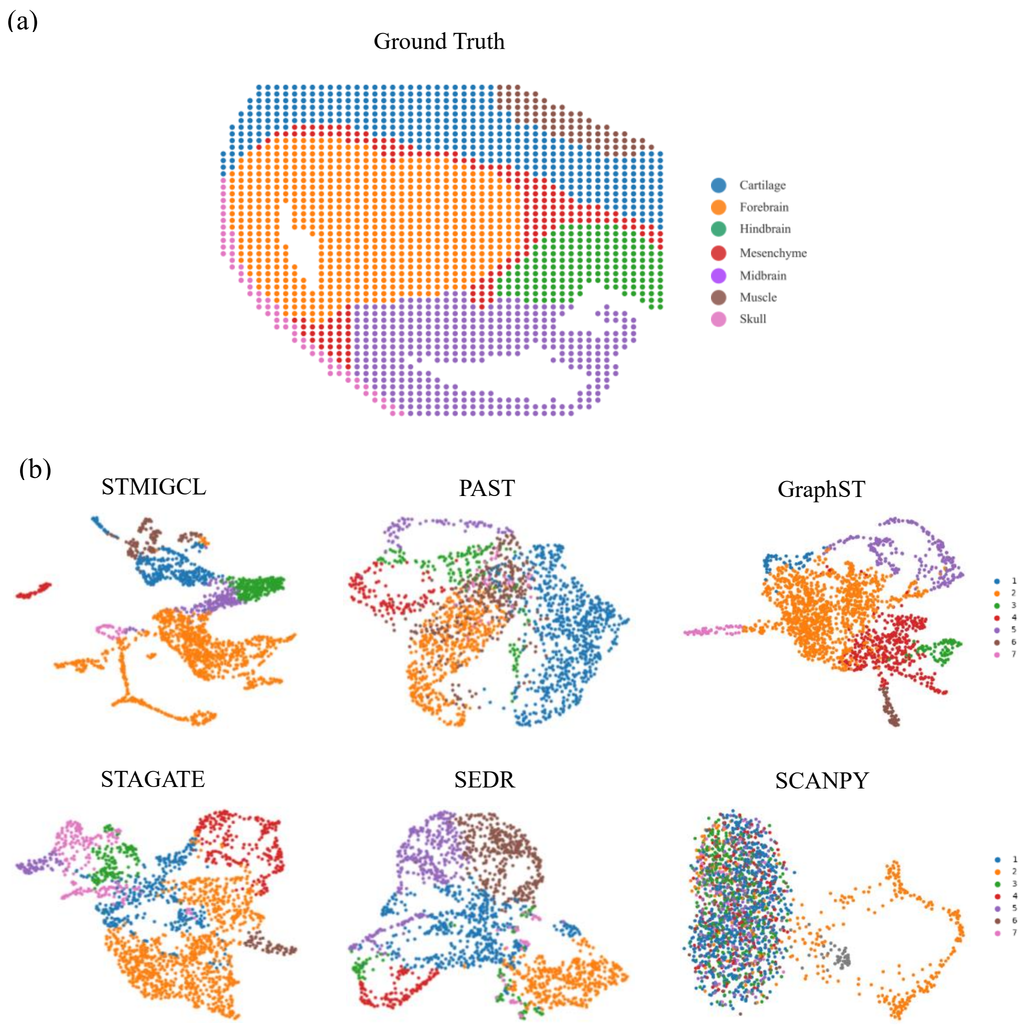

Figure S7. Application of STMIGCL to the spatial ATAC-seq data. (a) Manually annotated labels of mouse embryonic (E15.5) brain tissues in the MISAR-seq dataset. (b) 2D UMAP visualization of latent representations of ATAC data learned by STMIGCL, PAST, GraphST, STAGATE, SEDR and SCANPY, with colors denoting manually annotated labels.

3. Supplementary Tables

Table S1. Summary of the datasets used in our study

Platform	Tissue	Section	Spots	Genes	Reference
10x Visium	Human dorsolateral prefrontal cortex (DLPFC)	151507	4226	33538	[10]
		151508	4384		
		151509	4789		
		151510	4634		
		151669	3661		
		151670	3498		
		151671	4110		
		151672	4015		
		151673	3639		
		151674	3673		
		151675	3592		
		151676	3460		
	Human breast cancer	Human Breast Cancer Section 1	3798	36601	10x Visium demo
Stereo-seq	Mouse olfactory bulb	Puck_200127_15	19109	27106	[11]
	Mouse embryo	E9.5	5913	25568	[11]
STARMAP	Mouse visual cortex	X	1207	1020	[12]
osmFISH	Mouse somatosensory cortex	X	4839	33	[13]
spatial ATAC-seq	Mouse embryonic brain tissues	E15.5	1949	47287	[14]

Table S2. The quantitative evaluation results of STMIGCL and baseline methods on the Stereo-seq dataset of E9.5 mouse embryos

Methods	ARI Value	NMI Value
STMIGCL	0.58	0.64
GraphST	0.30	0.54
STAGATE	0.32	0.56
SEDR	0.30	0.54

Table S3. Performance variations on DLPFC dataset by using the three variants of STMIGCL to train the model

Slice	STMIGCL		w/o-F		w/o-S		w/o-A	
	ARI	NMI	ARI	NMI	ARI	NMI	ARI	NMI
151507	0.6194	0.7426	0.5697	0.7108	0.3651	0.5205	0.5249	0.6891
151508	0.5763	0.7018	0.5367	0.6776	0.3522	0.4952	0.5295	0.6401
151509	0.5365	0.6860	0.4660	0.6426	0.3586	0.5231	0.4424	0.6304
151510	0.4701	0.6614	0.4479	0.6439	0.2634	0.4372	0.4133	0.6079
151669	0.3757	0.5904	0.3020	0.4822	0.2760	0.4244	0.3137	0.4833
151670	0.4167	0.6092	0.3698	0.5983	0.3006	0.4572	0.2859	0.5534
151671	0.6037	0.7341	0.5261	0.6674	0.4591	0.5404	0.5507	0.6610
151672	0.6059	0.7229	0.5797	0.6688	0.3995	0.4945	0.4288	0.5589
151673	0.5979	0.7031	0.3921	0.5032	0.3781	0.4994	0.5257	0.6707
151674	0.5722	0.7230	0.4254	0.5473	0.3316	0.4615	0.5733	0.7140
151675	0.6170	0.7000	0.5101	0.6693	0.3715	0.4635	0.5325	0.6710
151676	0.5784	0.6684	0.4366	0.5754	0.3554	0.4615	0.4908	0.6115

Table S4. Setting of parameters for the STMIGCL

dataset	sample	npca	nemb	ncluster
Human dorsolateral prefrontal cortex	100	30	15	7 or 5
Human breast cancer	300	50	50	20
Mouse olfactory bulb	300	30	30	—
Mouse embryo	500	100	100	12
Mouse visual cortex	300	15	15	7
Mouse somatosensory cortex	500	30	30	11
Mouse embryonic brain tissues	500	30	30	7

References

[1] F. A. Wolf, P. Angerer, F. J. Theis, *Genome Biol* **2018**, *19*, 15.

[2] B. Wang, J. Luo, Y. Liu, W. Shi, Z. Xiong, C. Shen, Y. Long, *Briefings in Bioinformatics* **2023**, bbad262.

[3] Z. Li, X. Chen, X. Zhang, R. Jiang, S. Chen, *Genome Res.* **2023**, *33*, 1757.

[4] Y. Long, K. S. Ang, M. Li, K. L. K. Chong, R. Sethi, C. Zhong, H. Xu, Z. Ong, K. Sachaphibulkij, A. Chen, L. Zeng, H. Fu, M. Wu, L. H. K. Lim, L. Liu, J. Chen, *Nat Commun* **2023**, *14*, 1155.

[5] K. Dong, S. Zhang, *Nat Commun* **2022**, *13*, 1739.

[6] J. Li, S. Chen, X. Pan, Y. Yuan, H.-B. Shen, *Nat Comput Sci* **2022**, *2*, 399.

[7] E. Zhao, M. R. Stone, X. Ren, J. Guenthoer, K. S. Smythe, T. Pulliam, S. R. Williams, C. R. Uytingco, S. E. B. Taylor, P. Nghiem, J. H. Bielas, R. Gottardo, *Nat Biotechnol* **2021**, *39*, 1375.

[8] J. Hu, X. Li, K. Coleman, A. Schroeder, N. Ma, D. J. Irwin, E. B. Lee, R. T. Shinohara, M. Li, *Nat Methods* **2021**, *18*, 1342.

[9] H. Xu, H. Fu, Y. Long, K. S. Ang, R. Sethi, K. Chong, M. Li, R. Uddamvathanak, H. K. Lee, J. Ling, A. Chen, L. Shao, L. Liu, J. Chen, *Genome Med* **2024**, *16*, 12.

[10] K. R. Maynard, L. Collado-Torres, L. M. Weber, C. Uytingco, B. K. Barry, S. R. Williams, J. L. Catallini, M. N. Tran, Z. Besich, M. Tippani, J. Chew, Y. Yin, J. E. Kleinman, T. M. Hyde, N. Rao, S. C. Hicks, K. Martinowich, A. E. Jaffe, *Nat Neurosci* **2021**, *24*, 425.

[11] A. Chen, S. Liao, M. Cheng, K. Ma, L. Wu, Y. Lai, X. Qiu, J. Yang, J. Xu, S. Hao, X. Wang, H. Lu, X. Chen, X. Liu, X. Huang, Z. Li, Y. Hong, Y. Jiang, J. Peng, S. Liu, M. Shen, C. Liu, Q. Li, Y. Yuan, X. Wei, H. Zheng, W. Feng, Z. Wang, Y. Liu, Z. Wang, Y. Yang, H. Xiang, L. Han, B. Qin, P. Guo, G. Lai, P. Muñoz-Cánoves, P. H. Maxwell, J. P. Thiery, Q.-F. Wu, F. Zhao, B. Chen, M. Li, X. Dai, S. Wang, H. Kuang, J. Hui, L. Wang, J.-F. Fei, O. Wang, X. Wei, H. Lu, B. Wang, S. Liu, Y. Gu, M. Ni, W. Zhang, F. Mu, Y. Yin, H. Yang, M. Lisby, R. J. Cornall, J. Mulder, M. Uhlén, M. A. Esteban, Y. Li, L. Liu, X. Xu, J. Wang, *Cell* **2022**, *185*, 1777.

[12] X. Wang, W. E. Allen, M. A. Wright, E. L. Sylwestrak, N. Samusik, S. Vesuna, K. Evans, C. Liu, C. Ramakrishnan, J. Liu, G. P. Nolan, F.-A. Bava, K. Deisseroth, *Science* **2018**, *361*, eaat5691.

[13] S. Codeluppi, L. E. Borm, A. Zeisel, G. La Manno, J. A. van Lunteren, C. I. Svensson, S. Linnarsson, *Nat Methods* **2018**, *15*, 932.

[14] T. Tian, J. Zhang, X. Lin, Z. Wei, H. Hakonarson, *Nat Methods* **2024**, *21*, 1501.
